# Phenotypic Plasticity and Effects of Selection on Cell Division Symmetry in *Escherichia coli*

**DOI:** 10.1371/journal.pone.0014516

**Published:** 2011-01-10

**Authors:** Uttara N. Lele, Ulfat I. Baig, Milind G. Watve

**Affiliations:** 1 Department of Microbiology, Abasaheb Garware College, Pune, India; 2 Indian Institute of Science, Education, and Research, Pune, India; University of Washington, United States of America

## Abstract

Aging has been demonstrated in unicellular organisms and is presumably due to asymmetric distribution of damaged proteins and other components during cell division. Whether the asymmetry-induced aging is inevitable or an adaptive and adaptable response is debated. Although asymmetric division leads to aging and death of some cells, it increases the effective growth rate of the population as shown by theoretical and empirical studies. Mathematical models predict on the other hand, that if the cells divide symmetrically, cellular aging may be delayed or absent, growth rate will be reduced but growth yield will increase at optimum repair rates. Therefore in nutritionally dilute (oligotrophic) environments, where growth yield may be more critical for survival, symmetric division may get selected. These predictions have not been empirically tested so far. We report here that *Escherichia coli* grown in oligotrophic environments had greater morphological and functional symmetry in cell division. Both phenotypic plasticity and genetic selection appeared to shape cell division time asymmetry but plasticity was lost on prolonged selection. Lineages selected on high nutrient concentration showed greater frequency of presumably old or dead cells. Further, there was a negative correlation between cell division time asymmetry and growth yield but there was no significant correlation between asymmetry and growth rate. The results suggest that cellular aging driven by asymmetric division may not be hardwired but shows substantial plasticity as well as evolvability in response to the nutritional environment.

## Introduction

Aging is known to occur in bacteria and yeast that divide with morphological asymmetry [1]–[3]. For species that appear to divide symmetrically, aging was demonstrated relatively recently [4] and is thought to arise from aggregation and asymmetric inheritance of oxidatively damaged proteins [5]–[9]. In fact, the two cells after division can be viewed as a parent cell and a daughter cell rather than as two sister cells. This gives rise to a population dynamics that is similar to multicellular organisms that show age structured populations. Based on a Leslie Matrix model Watve et al. [10] simulated symmetric and asymmetric division and its effects on population growth. Their model assumed that the efficiency of cellular components declined with age and growth rate of a cell was a function of the relative proportions of new and old components. In asymmetrically dividing cells although a proportion of cells accumulated older components and ultimately died, young cells were being continuously generated and therefore the growth rate of the population remained high. On the other hand, in a symmetrically dividing culture, all the cells retained a proportion of older components resulting in slower growth of the entire population. Other studies based on mathematical models agreed on the growth rate advantage of asymmetric division [11]–[12]. In the Watve et al [10] model, at an optimum rate of repair the growth yield or biomass conversion efficiency of symmetrically dividing cells was predicted to be higher than asymmetrically dividing ones. Based on the simulation results Watve et al. [10] argued that symmetric division was a better strategy under low nutrient conditions when biomass conversion efficiency would be more critical. On the other hand under a nutritionally rich but highly competitive environment, asymmetrically dividing cells would gain a reproductive advantage. Another critical outcome of the model was that in low nutrient environments repair rates should be higher. Efficient repair systems can give fitness advantages only if the division is symmetric. Asymmetric division strategy on the other hand is unable to make optimum use of the repair mechanisms. The advantage of asymmetric division in increasing the growth rate has been demonstrated experimentally [4], [7]. The other prediction of the model, however, remains to be tested. If bacteria are shown to have different cell division asymmetries in different environments it would indicate that asymmetric division and aging may not be inevitable for bacteria but are adaptations for faster growth in a growth supportive and competitive environment.

A shift in cell division strategy, if any, may be observable in ecological or evolutionary time. If the cells have phenotypic plasticity, they may change cell division strategies in response to the environment in a short duration. Alternatively optimum cell division symmetry may evolve by prolonged selection in a given environment. Such a change should be observable in evolutionary time. We test here whether the asymmetry in cell division changes in response to the nutritional environment as per the model prediction [10] and whether this happens owing to phenotypic plasticity or genetic selection.

In rod shaped organisms it is possible that the morphologically old pole cell retains the damaged components [4], [5], [7]. However, this poses a potential paradox. If old and new poles were primarily associated with aging, spherical organisms such as *Staphylococcus* or *Micrococcus sp.* that divide in different planes and do not have definite poles would be immune to aging [13]. The association of asymmetry with polarity therefore needs to be examined in phylogenetic and ecological contexts. Even in earlier studies, in a significant proportion of cells, slower growth and protein aggregation is not necessarily associated with old poles [5], [7]. It is necessary to examine whether polarity is equally important in different strains of *Escherichia coli*. In the last five years since the demonstration of asymmetric division in *E. coli* [4] much research is focussed on the mechanisms of asymmetric segregation. Little progress is seen with regards to the eco-physiology and evolutionary angles of the problem. We address these issues by subjecting *E. coli* to selection under different sets of conditions and quantitatively examining the changes, if any, in cell division symmetry.

We cultured three strains of *E. coli* (*E. coli* ATCC 113-3D, *E. coli* KL16 and *E. coli* 2563 obtained from National Collection of Industrial Microorganisms, NCL Pune, referred hereafter as strains 1, 2 and 3 respectively) continuously under two sets of nutritionally extreme (high and low nutrient concentrations) conditions. Using 10 mg/ml and 0.1 mg/ml glucose as the sole source of carbon and energy, agar cultures were serially transferred up to an estimated 2000 cell generations such that at the end of the prolonged subculturing we had nine cell lineages, three selected for either of the nutritional environments and three wild types. In order to keep the temporal variation in substrate concentration to a minimum, the cultures were transferred frequently to ensure that they did not experience prolonged stationary phase or starvation any time. We denote the strain selected under high caloric environment as *H* and the one selected under low caloric environment as *L*. The current medium condition for the experiment is denoted by small letters *h* and *l* for high and low caloric conditions respectively and the subscript denotes original strain (e.g. strain 1 selected under high concentration but currently being grown in low concentration is denoted as *H_1_l*). After an estimated 1000 and 2000 generations of selection each of the lineages was examined for growth rate, growth yield and symmetry of cell division time on both high and low nutrient media.

## Results

### Index of cell division time asymmetry

**Figure 1:**
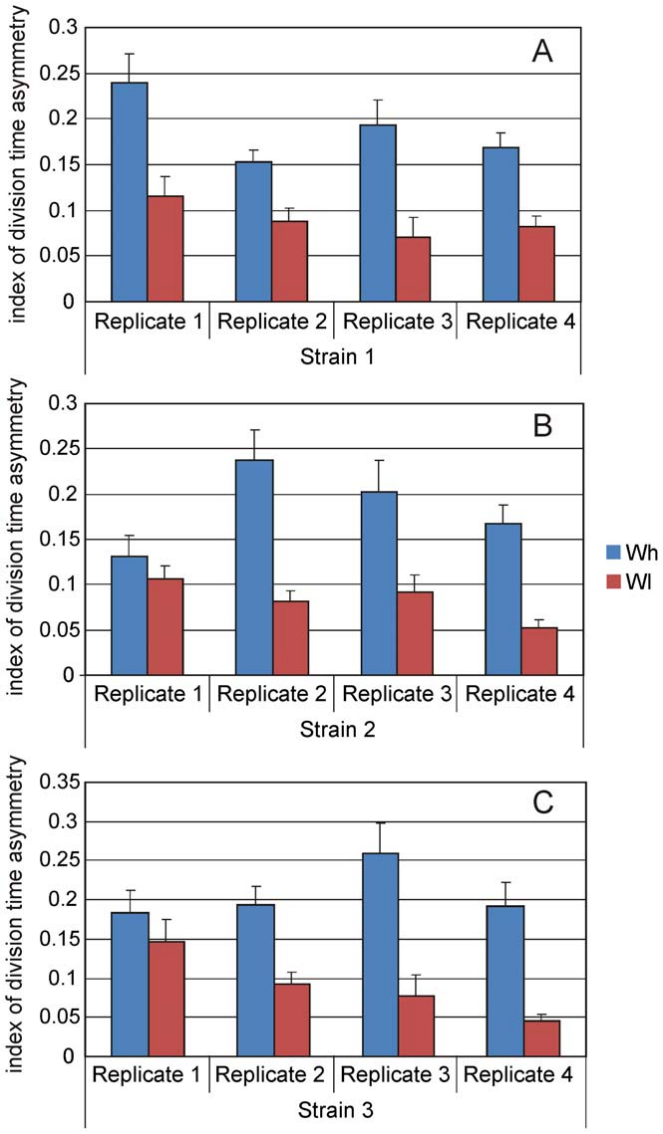
Glucose concentration and division time asymmetry. Effect of high and low glucose concentration (10 mg/ml and 0.1mg/ml respectively) on the mean index of division time asymmetry of all the wild strains is shown. Error bars represent s.e. Out of twelve replicates of three strains (A to C respectively), ten replicates showed significantly higher index of division time asymmetry under high glucose concentration than under low glucose concentration (see Table S1 for Mann Whitney results). This indicates plasticity in cell division time asymmetry and sensitivity to nutritional environment.

For quantifying functional symmetry in cell division we defined an index of cell division time asymmetry based on the assumption that if the cell division was asymmetric, the daughter cell receiving older components will take longer to complete the next cell division [4], [5], [7]. Therefore an index of asymmetry was defined as the ratio of the difference in division time (sign ignored) of two sister cells as to the average division time of the two. The index returns a value of zero if both sister cells complete their next division cycle at the same time. If the division of the slower of the two cells takes twice as much as the faster one the index takes a value of 2/3 and if it the difference is three fold the index becomes 1. The index is independent of the absolute time of division and does not reflect absolute growth rate. For all the three wild types, the cell division time asymmetry was measured under high and low nutrient concentrations in four replicates. In all the 12 experiments the index of asymmetry was greater under high nutrient concentration as compared to low (Figure 1A–C) and 10 out of the 12 differences were statistically significant (Mann Whitney U-test, see Table S1 for statistical details). This indicates that *E. coli* cell division symmetry is responsive to current nutritional environment and greater symmetry is exhibited under nutritional limitation.

**Figure 2:**
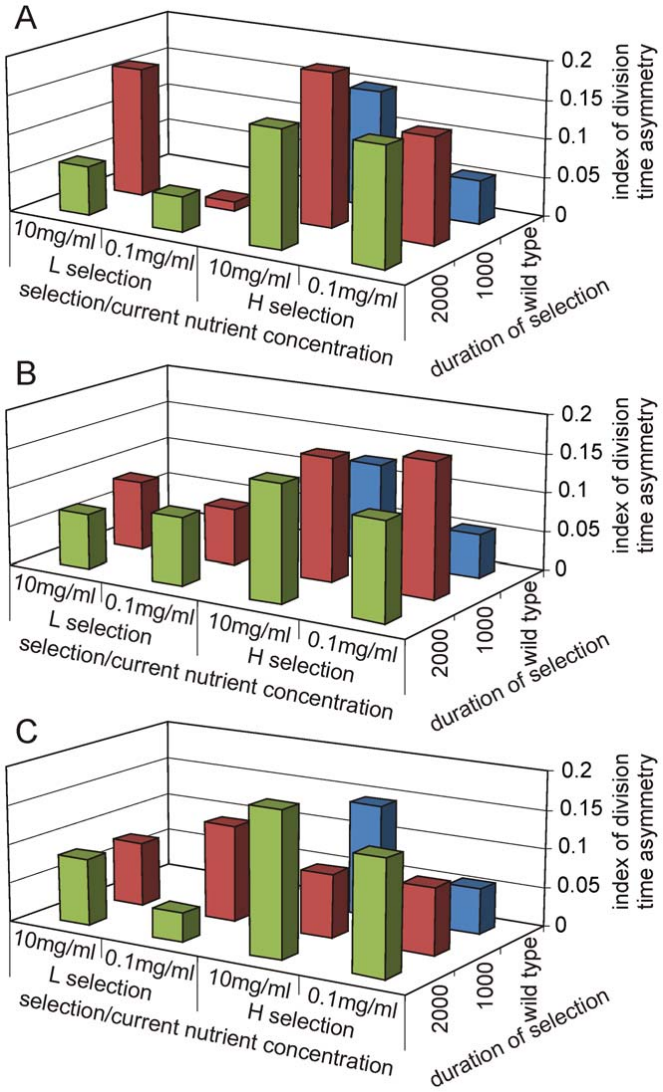
Division time asymmetry as a function of selection and current glucose concentration. Effect on the median index of cell division time asymmetry of current glucose concentration (10mg/ml and 0.1 mg/ml) and selection for 1000 and 2000 generations under high (H) and low (L) nutrient concentrations. A, B and C show results of strain 1, 2 and 3 respectively. Trends in means were similar. On prolonged selection *H* lineages develop consistently higher value of the index and lose sensitivity to the immediate nutritional environment. On the other hand, two of the three *L* lineages appear to be locked into lower asymmetry and lose responsiveness to environment.

For each of the strains the indices of cell division time asymmetry were calculated after selection for an estimated 1000 and 2000 generations. With strain 1 at 1000 generations, *H_1_l* had significantly higher asymmetry than *W_1_l* (p = 0.006) and *L_1_l* (p<0.0001), and *L_1_l* divided significantly more symmetrically than *W_1_l* (p = 0.001) (Figure 2A). *L_1_h* was significantly asymmetric than *L_1_l* similar to the wild type (p<0.0001). On the other hand, the asymmetry index of *H_1_h* was significantly greater than *H_1_l* only in a one tailed but not in two tailed test (one tailed p = 0.034). Further after 2000 generations of selection *H_1_l_2000_* did not differ significantly from *H_1_l_1000_* but the difference between *H_1_l* and *H_1_h* became non-significant. On the other hand asymmetry index of *L_1_h_2000_* decreased significantly from *L_1_h_1000_* (p<0.0001) and became indistinguishable from *L_1_l_2000_*. At 2000 generations, *H_1_h* was significantly greater than *L_1_h* (p<0.0001) and *L_1_l* (p<0.0001). Also *L_1_l* was significantly lower than *H_1_l* (p<0.0001) (Figure 2A). This indicates that although the wild type showed different asymmetry indices under *h* and *l*, the selected strains retained their symmetry or asymmetry even after changing current nutrient concentrations. In other words, *H_1_* became committed to asymmetric division and *L_1_* became committed to symmetric division with selection. In a pair-wise comparison at two substrate concentrations and two selection durations, all *L* strains had lower median asymmetry indices than their respective *H* counterparts (paired t = 4.287, p = 0.024).

The pattern of changes with selection was similar in strain 2. At 1000 generations, *H_2_h* divided significantly more asymmetrically than *L_2_l* (p<0.0001) and *W_2_h* (p = 0.03). The mean index of asymmetry of *L_2_h* was found to be significantly greater than *L_2_l* (p<0.0001) but *H_2_h* and *H_2_l* did not differ significantly in their asymmetry indices (Figure 2B). After 2000 generations *H_2_l_2000_* did not differ significantly from *H_2_l_1000_* and remained significantly greater than *L_2_l* (p = 0.001). *L_2_h_2000_* decreased significantly than *L_2_h_1000_* (p = 0.016) and showed significantly more symmetric division than *H_2_h* (p = 0.005) (Figure 2B). There was no significant difference in *H2h*, *H2L* and *L2l* between 1000 and 2000 generations. This indicates loss of plasticity with selection and commitment to symmetric division in *L_2_* and asymmetric division in *H_2_*. In a pair-wise comparison at two substrate concentrations and two selection durations, all *L* strains had lower median asymmetry indices than their respective *H* counterparts (paired t = 3.332, p = 0.044).

In strain 3 after 1000 generations *L_3_l* showed significantly lower asymmetry index than *L_3_h* (p<0.0001) but contrary to our expectations *L_3_h* showed significantly greater asymmetry than *H_3_h* (p = 0.049). No other differences were significant (Figure 2C). At 2000 generations, *L_3_h* (p = 0.004) and *H_3_l* (p<0.0001) showed significantly higher asymmetry than *L_3_l* (Figure 2C). There was no significant difference in asymmetries of *L_3_h*, *H_3_h* and *H_3_l*. Thus after prolonged selection *H_3_* appears to have committed to asymmetric cell division but *L_3_* unlike strain 1 and 2 did not commit to symmetric cell division (Table S1 and Dataset S1). In a pair-wise comparison at two substrate concentrations and two selection durations, unlike strains 1 and 2 *L_3_* strains did not differ significantly than their respective *H_3_* counterparts (paired t = 0.663, p = 0.554). Pooling data on all the three strains, the *L* lineages had significantly lower median asymmetry indices than their *H* counterparts (paired t = 3.345, p = 0.006).

Taking the median indices of all the three strains at both selection conditions, selection durations and current nutrient concentrations we performed a four factor ANOVA without replication which revealed that only the effect of selection was statistically significant (p = 0.05) whereas strain, duration of selection and current nutrient concentration did not affect asymmetry index significantly. In the wild type, the effect of current nutrient concentration was highly significant whereas after selection it appears to have lost its significance in most of the pairs. This indicates that selection led to commitment to one type of division and the plasticity of the response was significantly reduced. This change was evident by 1000 generations itself but appeared to become more pronounced by 2000 generations with the only exception of *L_3_*.

The proportion of cells that become exceedingly slow are represented as outliers in the distribution of asymmetry index. Some of the cells failed to divide until the end of the observation period, owing to which an index of asymmetry for such pairs could not be calculated. However their index can be assumed to be greater than the one calculated taking the end of observation period as the division time. Extreme outliers in the distribution, including the cells that failed to divide were assumed to be old or dead cells. Since the frequency of such outliers is small, comparisons according to selection-treatment-strain wise categories was not statistically meaningful. Therefore we compared the frequency of outliers by pooling data. Taking a cut off asymmetry index of 0.667 (corresponding to old cell having at least double the division time of the young sibling) to define old cells, all *H* lineages pooled together had significantly greater proportion of old cells (0.051) than all wild types pooled (0.019) and all *L* lineages pooled (0.005) (*Z* test for comparison of proportions, one-tailed, p = 0.01 and <0.001 respectively). The difference between old cells in the wild type and *L* lineages was also significant (p = 0.04). Current concentration of medium did not show a significant difference although the difference was in the expected direction (all *h* pooled 0.033, all *l* pooled 0.019, p>0.05). Comparing the three strains, strain 2 (all selection-treatment pooled) had significantly greater proportion of old cells than the other strains (Monte Carlo method for comparison of more than 2 proportions, frequencies of old cells 0.010, 0.0455 and 0.023 respectively for strain 1, 2 and 3, p = 0.001).

### Effect of selection on cell morphology

It can be argued that the cell division time asymmetry may be a result of stochastic morphological asymmetry. If the two daughter cells have a difference in cell length, the shorter of the two cells may take a longer time to grow and undergo next division. We therefore examined morphological asymmetry and its relation to time asymmetry. Morphological asymmetry index was defined as the difference in the lengths of the two sister cells divided by pre-division length of the mother cell. For all the three strains with and without selection, the division was morphologically more symmetrical in low nutrient concentration than in high (Dataset S2). However there was no significant correlation between morphological asymmetry and division time asymmetry indicating that morphological asymmetry was unlikely to be the cause of division time asymmetry. As a result of selection cell lengths significantly increased in *H_1_* (p<0.0001), *H_2_* (p = 0.002) and *H_3_* (p<0.0001) whereas they remained indistinguishable from the wild types in *L_1_*, *L_2_* and *L_3_*.

### Growth rates and growth yields

**Figure 3:**
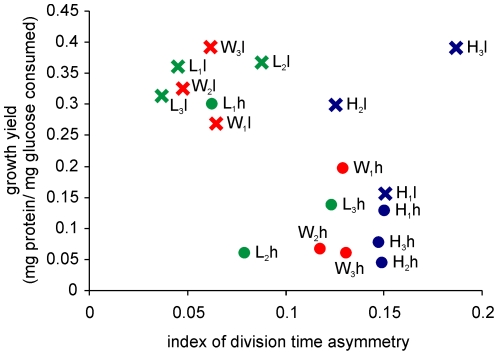
Growth yield and index of division time asymmetry. Significant negative correlation was seen between growth yields and indices of asymmetry in the pooled data (τ = −0.320, p = 0.031). Wild types are shown in red, *H* in blue and *L* in green. Crosses indicate low current concentration and solid circles high current concentration.

**Figure 4:**
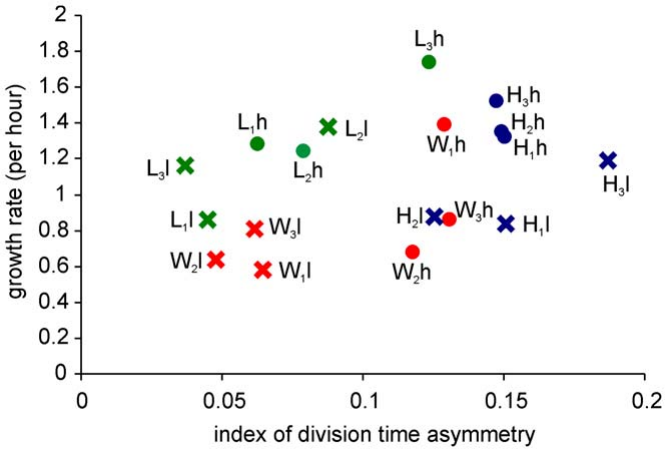
Cell division time asymmetry index and growth rate. No significant correlation was seen between cell division time asymmetry indices and growth rates. (τ = 0.210, p = 0.112). Colour codes same as figure 3.

**Figure 5:**
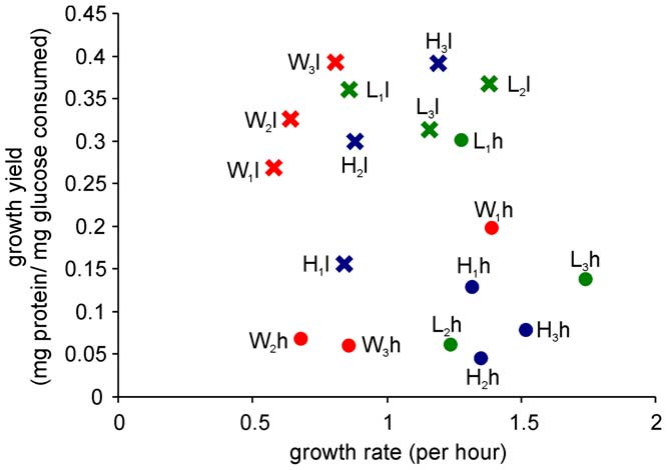
Growth yield and growth rate of the selected and wild strains. No significant correlation was seen between growth yields and growth rates. (τ = −0.157, p = 0.181). Colour codes same as figure 3.

As the Watve et al [10] Model predicted, asymmetric division is expected to increase growth rates and symmetric cell division may have an advantage of higher biomass conversion rates. Although the selection and monitoring of cell division symmetries was done on agar media, owing to the difficulty of calculating growth yields on agar media the growth rate and growth yield estimations were performed in shake flask cultures in liquid media. All the three wild type strains gave higher growth yields in *l* as compared to *h* but the growth rates were indistinguishable for two of the three strains. For strain 1 growth rate was higher in *h* as compared to *l*. In two of the three strains the growth rates increased as a result of selection both under low and high glucose concentration. In strain 1 the growth rate increased during selection in low substrate concentration but decreased in high substrate concentration. The correlation between index of asymmetry and growth yield for all the selected and wild type lines of all the three strains in both the nutrient conditions was found to be significantly negative in agreement with the model prediction (Figure 3) (τ = −0.320, p = 0.031). However contrary to the model prediction no significant correlation was seen in the index of asymmetry and growth rates (Figure 4). Also no significant negative correlation between growth rates and growth yields was found (Figure 5) unlike what the growth rate growth yield trade-off [14], [15] predicts.

### Cell polarity and division time asymmetry

To check if morphological old pole cell is also functionally old in all the lineages, we compared the relative time taken by the old pole cell and the new pole cell for the subsequent division. In a pair wise comparison pooling over all conditions only in strain 2 there was significant difference in the division time of the old pole cell as compared to the corresponding new pole cell (paired t-test one tailed: n = 297, n_1mean_ = 1.0147, n_2mean_ = 0.9853, p = 0.032). Segregating data according to treatments, in 12 out of 18 strain-treatment combinations, old poles cells divided slower than the new pole cells but only four of the differences were significant (*W_1_l*: n = 33, n_1mean_ = 1.026,n_2mean_ = 0.974, p = 0.04; *W_2_h*: n = 51, n_1mean_ = 1.047,n_2mean_ = 0.953, p = 0.036; *W_2_l*: n = 34, n_1mean_ = 1.021, n_2mean_ = 0.979, p = 0.045; *L_3_h*: n = 50, n_1mean_ = 1.026, n_2mean_ = 0.974, p = 0.032). In the remaining 6 a reversed pattern was seen and only one of the differences was significant (*W_3_h*: n = 42, n_1__mean_ = 0.968, n_2mean_ = 1.032, p = 0.03). These results indicate that the association of old pole with aging was only weakly and inconsistently significant in these lineages.

## Discussion

### Is cellular aging inevitable in *E. coli*?

Our experiments demonstrate that asymmetry in cell division is not hard wired but is responsive to environmental conditions. The wild types showed phenotypic plasticity in cell division symmetry before selection in response to high and low nutrient concentrations. It is important to note that despite this change the response to nutrient concentration remained the same demonstrating the robustness of this association. Prolonged selection in high nutrient concentrations induced commitment to asymmetric cell division in the cultures and selection in low nutrients caused commitment to symmetric cell division. This might reflect that there is a cost associated with phenotypic plasticity owing to which under a monotonous regime the plasticity was lost. A subtle difference between the two types of commitments was that commitment to asymmetric cell division was faster than commitment to symmetric cell division. At 1000 generations of selection all the three strains being selected under high nutrient concentration showed commitment to asymmetric division. The *L* lineages, on the other hand retained plasticity. After 2000 generations all the three *H* strains showed commitment to asymmetry but only two of the three *L* showed commitment to symmetric division. This may lead to a speculation that asymmetric division may be associated with a lower cost than symmetric division, although it may be too early to make such inferences.

In an experimental set up such as the above, it is difficult to define a dead cell. We assume cells that stop dividing during the observation period as presumably dead and cells that divide slowly as old. The former is particularly difficult to validate. However if we accept this as a working definition, the proportion of old and dead cells was greater in the *H* selected lineages that were committed to asymmetric division. This observation is compatible with the model prediction [10] that symmetric division may delay or avoid aging.

The results are compatible with the theoretical prediction [10] that asymmetric division and aging will evolve in nutrient rich environments and symmetric cell division and non-aging population may evolve in caloric restricted environments.

### Correlations with growth rates and growth yields

We did not find a positive correlation between growth rates and index of asymmetry as the model had predicted. Growth rates in high current concentrations were greater in 6 out of 9 strains where as they did not differ substantially in the remaining 3. It is evident therefore that the low nutrient concentration was growth limiting. It is interesting to note that even in the strains where growth rates in *h* and *l* did not differ, the asymmetry index was significantly different. This is suggestive of at least partial independence of the mechanisms to regulate growth rates and cell division symmetry. Also contrary to our expectations *H* selection did not give rise to strains with higher growth rates than *L* selection. As opposed to growth rates, Growth yields correlated negatively with the index of asymmetry consistent with the model prediction but we did not find the expected negative correlation between growth rates and growth yields. It appears therefore that although the model prediction about cell division symmetry was well supported by the experiments, the predictions about growth rate and growth yields were not quite well supported. However, caution is needed in inferring from these result. In the design of the experiment, cell divisions had to be monitored on agar surface since liquid cultures do not allow monitoring of the same cell over a long time, and growth yields could be estimated only in liquid media where even distribution of nutrients throughout the medium can be ensured. This inevitable flaw in the experiment could have led to certain artefacts. For example the growth parameters in liquid and solid media may be sufficiently different to distort the correlations. The inability to get different growth rates in *H* and *L* lines may be owing to the selection regime which selected for higher growth rates in either of the nutrient concentrations. Growth rates increased after subculturing in high as well as low nutrient concentrations and it is likely that other mechanisms facilitating growth rate might have dominated the results masking the effect of cell division symmetry on growth rate.

Symmetric cell division should be accompanied by the optimization of repair rates according to the Watve et al. [10] model. Our experiments could not test this prediction directly. However, in the model, increase in the growth yield accompanying symmetric cell division critically depended on optimization of repair or recycling rates. Therefore higher growth yield can be taken as an indirect evidence for higher repair rates accompanying symmetric cell division.

### Is the association more general?

It is well known that caloric restriction leads to longevity in widely differing organisms including yeast, *Caenorhabditis elegans*, *Drosophila*, and mammals although it cannot be said to be universal [16], [17]. The association between cell division symmetry and caloric restriction could be a more general phenomenon in unicellular organisms. Mutation in Sir2, a gene necessary for asymmetric segregation in yeast [8], resulted in a longer chronological life span under caloric restriction [18]. It appears that Sir2 activation, leading to asymmetric segregation, reduces the chronological life span in yeast [18], [19] although its role in reproductive life span extension is debated [19], [20]. Other indications such as higher growth yields in oligophilic bacteria [21], growth rate and growth yield trade off [14], [15] suggest that the relationship between caloric restriction, cell division symmetry, high repair rates and high growth yield could be more generally true and responsible for longevity in widely differing organisms.

Asymmetry of division may have comparable implications in unicellular and complex organisms. In unicellular organisms asymmetric division may have evolved to increase the growth rate of the population by continually generating young cells, where as in multicellular organisms asymmetric division seems to rejuvenate stem cells [22]–[25]. However, the consequences of symmetric division for aging may be different in unicellular and multicellular organisms. Caloric restriction induced symmetric division may arrest aging in unicellular organisms but this mechanism need not be relevant to aging in multicellular organisms. Instead enhanced repair rate in response to caloric restriction predicted by the model could be more relevant.

### Mechanisms of asymmetric segregation

Our studies do not address the question of how asymmetric segregation is achieved. However, considerable progress has been made in understanding how damaged proteins are asymmetrically segregated in daughter cells and protein aggregation appears to be one of the mechanisms of cell division asymmetry [5]–[7]. If protein aggregation is central to bacterial aging, the process of aggregation should also be flexible and responsive to environmental stimuli. Although protein aggregation has not been studied under variable nutritional environments, flexibility of the process in terms of reversibility and disaggregation has been demonstrated [26]. The Heat shock proteins play a role in protein aggregation and the expression of HSPs is known to be different in the stationary phase [27] as compared to the growth phase raising the possibility that protein aggregation may be responsive to the nutritive environment. There are other possible mechanisms too. Polyphosphate accumulation in response to nutrient stress has been shown to affect polar localization of proteins and this relocation response may bring greater symmetry in component distributions on starvation [28].

On the other hand it is questionable whether polar localization is central to aging in bacteria. In rod shaped bacteria including *E. coli* the poles and polarisation of protein aggregates may play an important role. But spherical bacteria that divide in more than one plane do not have distinct poles. If poles are indeed central to cellular aging, cocci should be immune to aging [13]. Currently there is no published data on cell division symmetry or aging in spherical bacteria. Our preliminary observations show cell division asymmetry in staphylococci (data not shown). Even in *E. coli* protein aggregates are not always associated with the old pole [5], [7]. Unlike Stewart et al [4] and Lindner et al [5], we find only marginal association of poles with cell division times. It is possible that the extent of aggregation rather than polarity plays a major role in asymmetric segregation and the extent of association of aggregates with old poles may be different in different strains.

An important issue in the context of selection is whether there is a cost involved in mechanisms of protein aggregation or aggregation is an entropy-driven process and its prevention or disaggregation needs extra cost. Contradictory claims have been made regarding the costs of aggregation and disaggregation [7], [26]. The cost will certainly influence the direction and strength of selective forces. Our finding that commitment to asymmetric division appears faster and more consistent suggests that there could be greater cost in symmetric division so that mutants that lack this costly facility rapidly get selected when it is not needed. It is necessary at this stage that research on the proximate causes (mechanisms) of asymmetric division and ultimate causes (differential selective advantages in different conditions) follow a coherent path.

## Materials and Methods

### Media compositions

Composition of Glucose mineral medium: Na_2_HPO_4_ 0.1 gm%, KNO_3_ 0.3 gm%, (NH_4_)_2_SO_4_ 0.05 gm%, NaCl 0.05 gm%. Glucose: For high concentration medium: 10 mg/ml; for dilute medium: 0.1 mg/ml. All media were prepared in distilled water and pH was adjusted to 7 and sterilized by autoclaving at 115°C for 20 minutes. For the observations of cell division slides of the media mentioned above were used with 2% agar. All incubations as well as microscopy was done at 28°C.

### Determination of growth rates and growth yields

Growth rates and growth yields were determined in liquid culture in triplicates as follows. Growth rates were calculated in shake flask cultures based on the difference in the log absorbance per unit time at mid-exponential growth phase when the slope of the growth curve was maximum. Growth yields were calculated by harvesting cells by centrifugation from 40 ml broth at the beginning of stationary phase. Total cell protein was estimated by Folin-Lowry method and initial and residual glucose was estimated by using Di-nitro Salicylic acid method. Growth yield was expressed as total cell protein produced per unit of glucose consumed.

### Selection under high and low nutrient concentrations

Continuous subculturing of all the cell lineages was done on agar plates with media composition mentioned above. An advantage of using solid medium was that any contaminant could be easily detected and avoided. The subculturing was done from 10–15 colonies. The number of generations per subculturing was estimated from the estimated mean number of cells per colony. On high nutrient concentration the estimated number of generations was 22 and on low nutrient concentration 19 to 20. For convenience of inoculation, one subculture prior to microscopy was done in liquid medium of the same composition. After 2000 generations of subculturing the 16S rDNA sequences of all lineages were matched with the wild types to confirm the identity.

### Microscopy and cell division observations

Photomicrography was used to observe cell division and developing microcolonies. A broth culture in its mid-exponential phase was poured on a sterile slide coated with agar medium with the same composition as the broth. Although the nutrient composition in broth and on the slide was the same and the cells were in mid exponential phase, a short lag after transferring from broth to slide was observed presumably owing to transfer from a liquid to a solid surface. After the lag subsequent divisions and microcolony development could be monitored by time lapse photomicrography. Time-lapse microscopy and imaging was performed to observe cell divisions in developing micro-colonies on Zeiss Imager (Axio Imager Z1) microscope with the magnification of 63× OEL objective and Differential Interference Contrast (DIC) technique in transmitted light till the microcolonies reached 16 to 32 cells stage, i e. 4 to 5 generations (Figure S1). For all the samples cell division time asymmetry indices on a minimum sample size of 45 pairs of sister cells with a maximum of 8 from a single microcolony were recorded. Cell lengths were measured using ImageJ software calibrated for Axio-Vision microscopy software.

### Experimental design

Three strains of *E. coli* (*E. coli* ATCC 113-3D, *E. coli* KL16 and *E. coli* 2563 obtained from National Collection of Industrial Microorganisms, NCL Pune, referred as strains 1, 2 and 3 respectively) were grown in liquid culture at high (10 mg/ml) and low (0.1 mg/ml) glucose concentrations and at mid-exponential growth phase used to inoculate slides for time-lapse microscopy as described above. The experiment was performed in four replicates and the index of division time asymmetry under high and low glucose concentrations was calculated. Each strain was subjected to the selection procedure as mentioned above on high and low nutrient concentrations simultaneously. At estimated 1000 and 2000 generations of selection, the selected strains were again tested for index of division time asymmetry on both the nutrient concentrations. At the end of 2000 generations growth rate and growth yield were determined again on both the nutrient concentrations.

### Statistical Analysis

The frequency distribution of cell division time asymmetry index was highly skewed in all the lineages. The trends in means and medians were similar. Ideally medians are preferred over means in skewed distributions. However since outliers in our data, which are old cells are central to the hypothesis being tested, we used means wherever important. Although the experiment had a factorial design, owing to the skewness of the data ANOVA could not be performed. We therefore relied on pair wise comparisons using nonparametric tools. Mann-Whitney test was used for comparison of asymmetry indices of any two lineages. For correlation analysis nonparametric Kendall's tau was used.

## Supporting Information

Table S1 Statistical analysis of index of cell division time asymmetry is given.
(0.05 MB DOC)

Dataset S1 Raw data on cell division time asymmetry indices.
(0.08 MB XLS)

Dataset S2 Record of cell length measurements during microcolony development.
(0.12 MB XLS)

Figure S1 Selected images during microcolony development.
(8.05 MB PPT)
