# Supplementary material for: Phenotypic Plasticity and Effects of Selection on Cell Division Symmetry in Escherichia coli
Source: PLoS One. 2011 Jan 10;6(1):e14516. doi: 10.1371/journal.pone.0014516 (PMC3018420; doi:10.1371/journal.pone.0014516)
Supplement: Table S1 — Statistical analysis of index of cell division time asymmetry is given. (0.05 MB DOC) [file pone.0014516.s001.doc]

**Table S1:**

**Statistical analysis of indices of cell division time asymmetry**

The frequency distribution of indices was found to be highly positively skewed and with extreme outliers. Therefore although the experimental design is factorial, ANOVA could not be performed using the complete data. At the level of individual strain-current substrate combinations, pair-wise comparisons were made using the non-parametric Mann-Whitney U test. Following table gives n1, n2, U and p-values respectively of all the tests performed. The disadvantage of such an approach is that since a large number of tests is being done, a proportion of the tests may turn out to be significant by chance alone. We therefore apply a Bonferroni correction to the significance level. These corrections make the test highly conservative. It can be seen that in spite of being highly conservative the main body of results retains its significance. Asterisks denote significance after Bonferroni correction.

|  | | **Strain 1** | | **Strain 2** | | **Strain 3** | |
| --- | --- | --- | --- | --- | --- | --- | --- |
| Wh &Wl | Replicate 1 | (53, 53) 2122 (<0.0001)* | | (25, 45) 644 (0.317) | | (27, 26) 404 (0.345) | |
|  | Replicate 2 | (59, 57) 2372 (<0.001)* | | (61, 61) 2634 (<0.0001)* | | (54, 56) 2179 (<0.0001)* | |
|  | Replicate 3 | (27, 28) 611 (<0.0001)* | | (30, 33) 698 (0.005) | | (28, 10) 283 (0.001) | |
|  | Replicate 4 | (45, 47) 1589 (<0.0001)* | | (38, 46) 1417 (<0.0001)* | | (41, 31) 1075 (<0.0001)* | |
|  | | **Strain 1** | **Strain 2** | **Strain 3** | **Strain 1** | **Strain 2** | **Strain 3** |
|  |  | At 1000 generations | | | At 2000 generations | | |
| Wh & Hh | | (53, 58)  1568  (0.855) | (25, 50)  817  (0.030) | (27, 30)  324  (0.195) | (59, 70)  2212  (0.487) | (63, 99)  3078  (0.889) | (54, 53)  1300  (0.416) |
| Wh & Hl | | (53, 53)  1758  (0.025) | (25, 37)  312  (0.031) | (27, 23)  408  (0.057) | (59, 78)  2153  (0.521) | (63, 68)  2105  (0.866) | (54, 56)  1360  (0.363) |
| Wh & Ll | | (53, 53)  1485  (<0.0001)* | (25, 87)  1291  (0.137) | (27, 44)  888  (<0.0001)* | (59, 65)  2766  (<0.0001)* | (63, 70)  2856  (0.003) | (54, 42)  1708  (<0.0001)* |
| Wh & Lh | | (53, 53)  2122  (0.611) | (25, 55)  576  (0.247) | (27, 49)  635  (0.765) | (59, 58)  1027  (<0.0001)* | (63, 43)  946  (0.009) | (54, 73)  1689  (0.169) |
| Wl & Hh | | (53, 58)  896  (<0.0001)* | (45, 50)  628  (<0.0001)* | (26, 30)  412  (0.711) | (57, 70)  2882  (<0.0001)* | (61, 99)  1722  (<0.0001)* | (56, 53)  964  (0.002) |
| Wl & Hl | | (53, 53)  1837  (0.006) | (45, 37)  420  (<0.0001)* | (26, 23)  342  (0.393) | (57, 78)  1208  (<0.0001)* | (61, 68)  3141  (<0.0001)* | (56, 56)  804  (<0.0001)* |
| Wl & Ll | | (53, 53)  885  (0.001) | (45, 87)  2089  (0.527) | (26, 44)  740  (0.030) | (57, 65)  1916  (0.742) | (61, 70)  2502  (0.086) | (56, 42)  1134  (0.760) |
| Wl & Lh | | (53, 53)  903  (0.002) | (45, 55)  859  (0.009) | (26, 49)  523  (0.204) | (57, 58)  1601  (0.769) | (61, 43)  1100  (0.159) | (56, 73)  1459  (0.005) |
| Hh & Hl | | (58, 53)  1845  (0.068) | (50, 37)  902  (0.843) | (30, 23)  370  (0.653) | (70, 78)  2746  (0.949) | (99, 68)  3245  (0.695) | (53, 56)  1216  (0.105) |
| Hh & Ll | | (58, 53)  513  (<0.0001)* | (50, 87)  3233  (<0.0001)* | (30, 44)  814  (0.088) | (70, 69)  3391  (<0.0001)* | (99, 70)  4500  (0.001) | (53, 42)  1547  (0.001) |
| Hh & Lh | | (58, 53)  1593  (0.741) | (50, 55)  1543  (0.281) | (30, 49)  540  (0.049) | (70, 58)  2930  (<0.0001)* | (99, 43)  2761  (0.005) | (53, 73)  2067  (0.511) |
| Hl & Ll | | (53, 53)  522  (<0.0001)* | (37, 87)  2434  (<0.0001)* | (23, 44)  405  (0.181) | (78, 65)  1286  (<0.0001)* | (68, 70)  3234  (<0.0001)* | (56, 42)  554  (<0.0001)* |
| Ll & Lh | | (53, 53)  553.5  (<0.0001)* | (87, 55)  3238  (<0.0001)* | (44, 49)  1660  (<0.0001)* | (65, 58)  1707  (0.364) | (70, 43)  1500  (0.979) | (42, 73)  1044  (0.004) |
