# Supplementary material for: Phenotypic Plasticity and Effects of Selection on Cell Division Symmetry in Escherichia coli
Source: PLoS One. 2011 Jan 10;6(1):e14516. doi: 10.1371/journal.pone.0014516 (PMC3018420; doi:10.1371/journal.pone.0014516)

## Slide 1
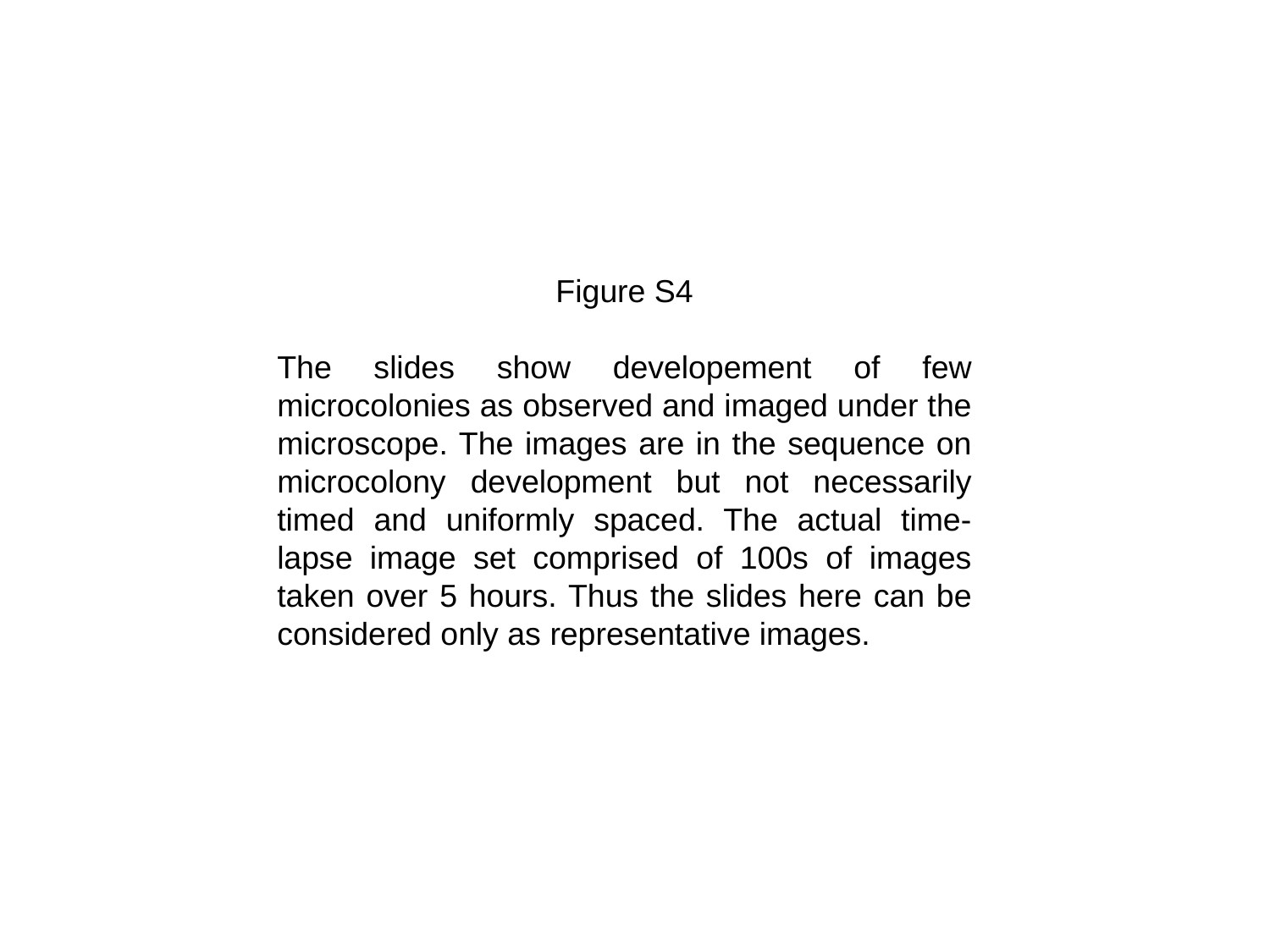

Figure S4
The slides show developement of few microcolonies as observed and imaged under the microscope. The images are in the sequence on microcolony development but not necessarily timed and uniformly spaced. The actual time-lapse image set comprised of 100s of images taken over 5 hours. Thus the slides here can be considered only as representative images.

## Slide 2
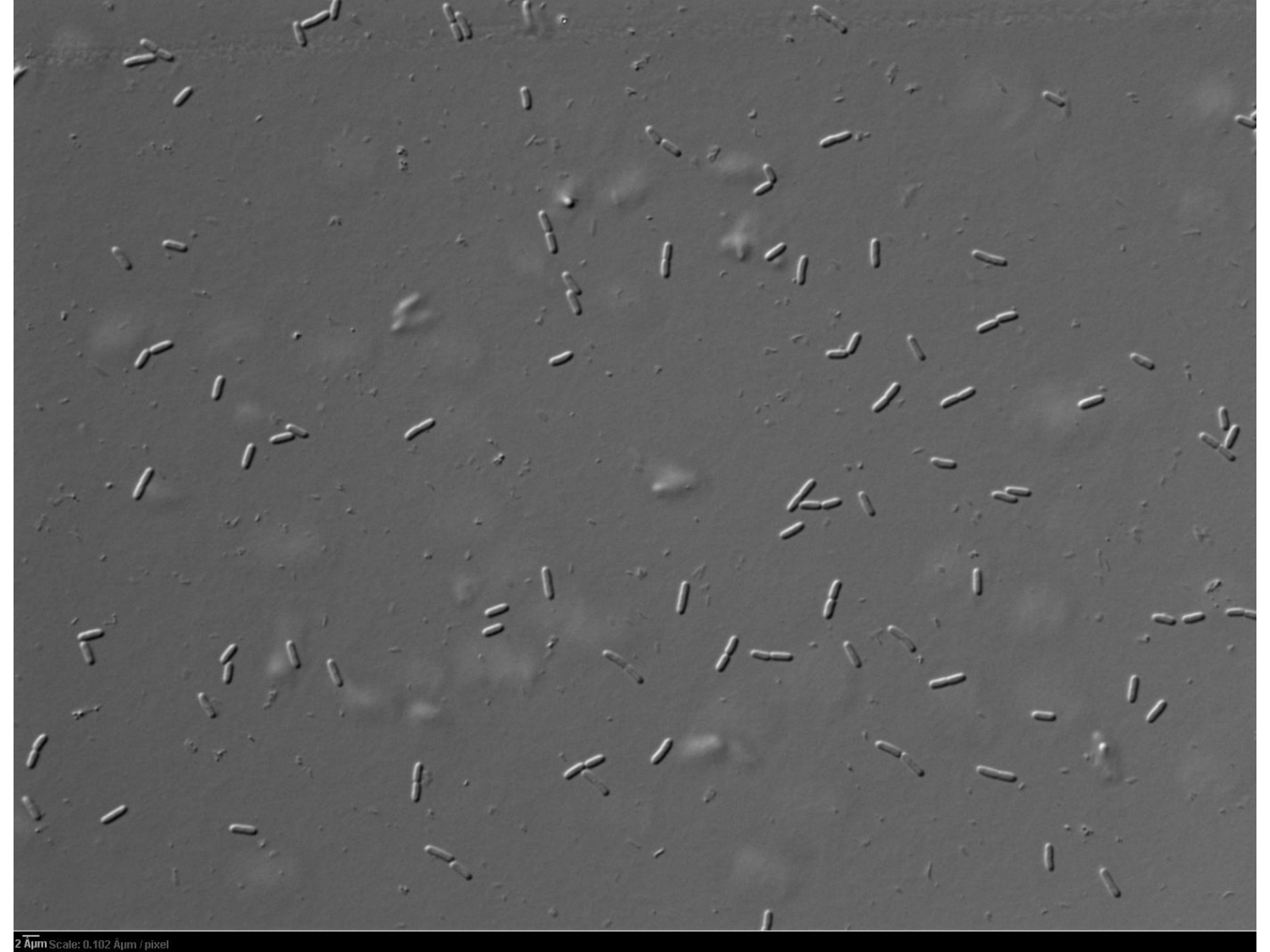

## Slide 3
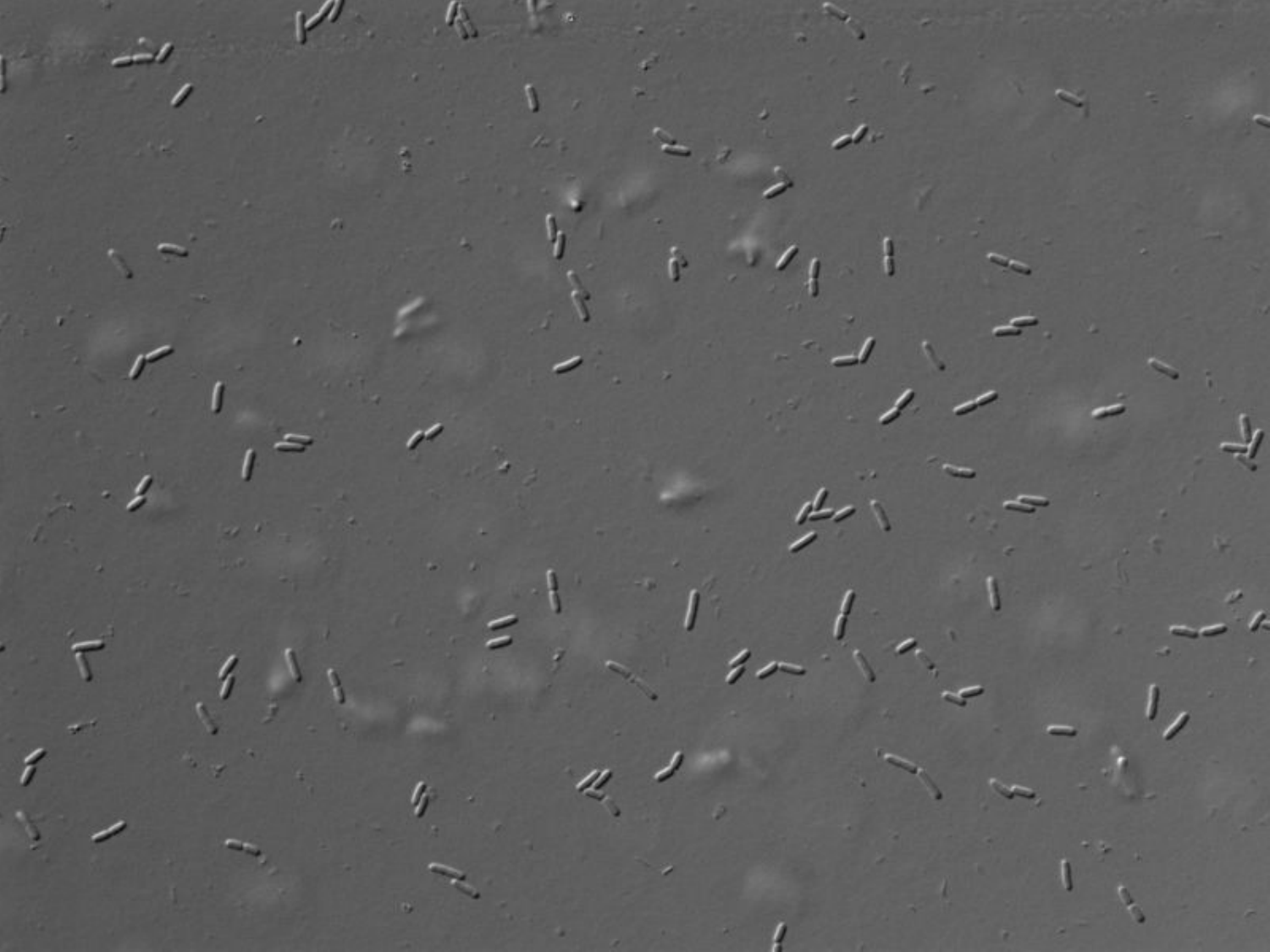

## Slide 4
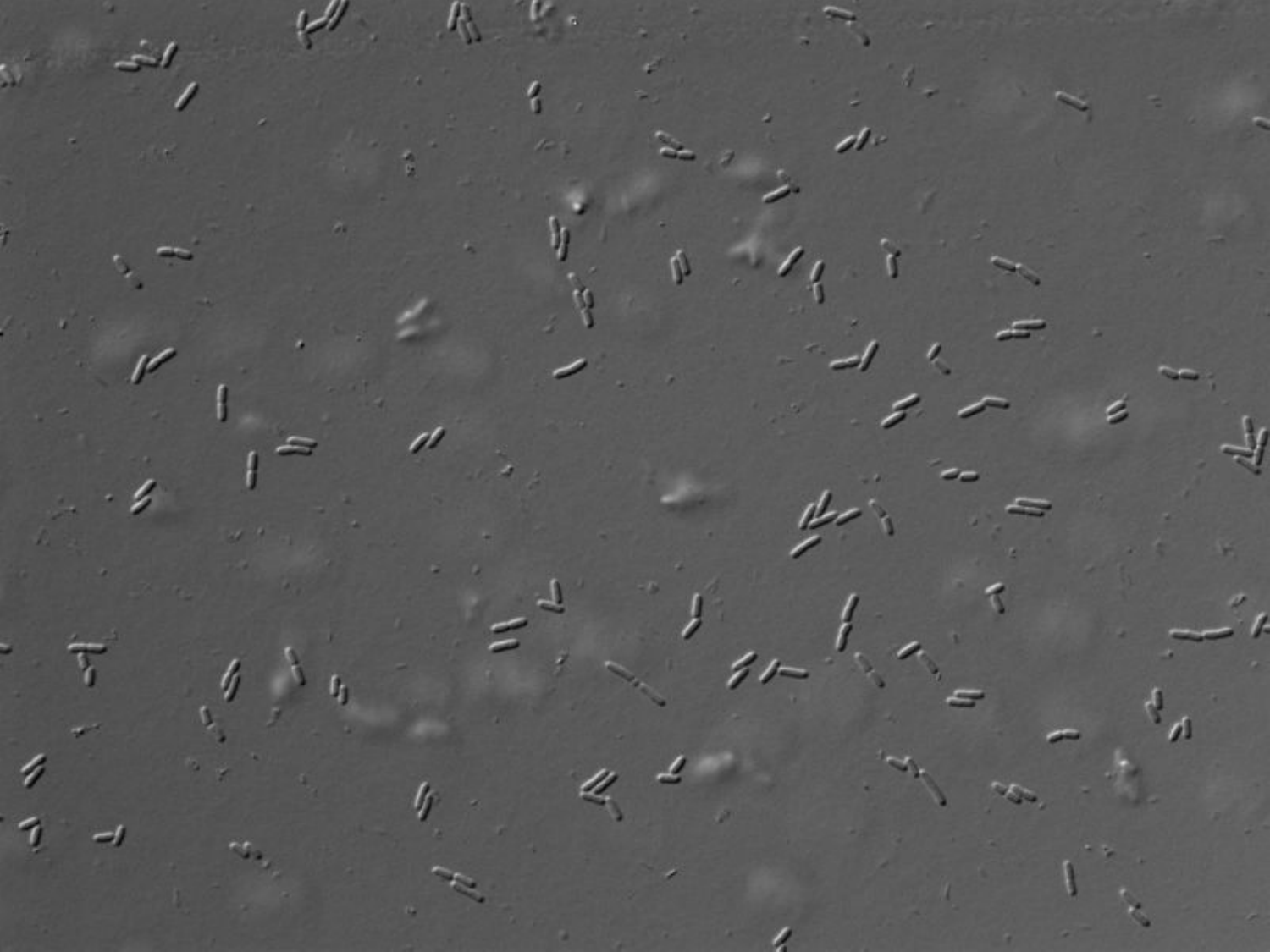

## Slide 5
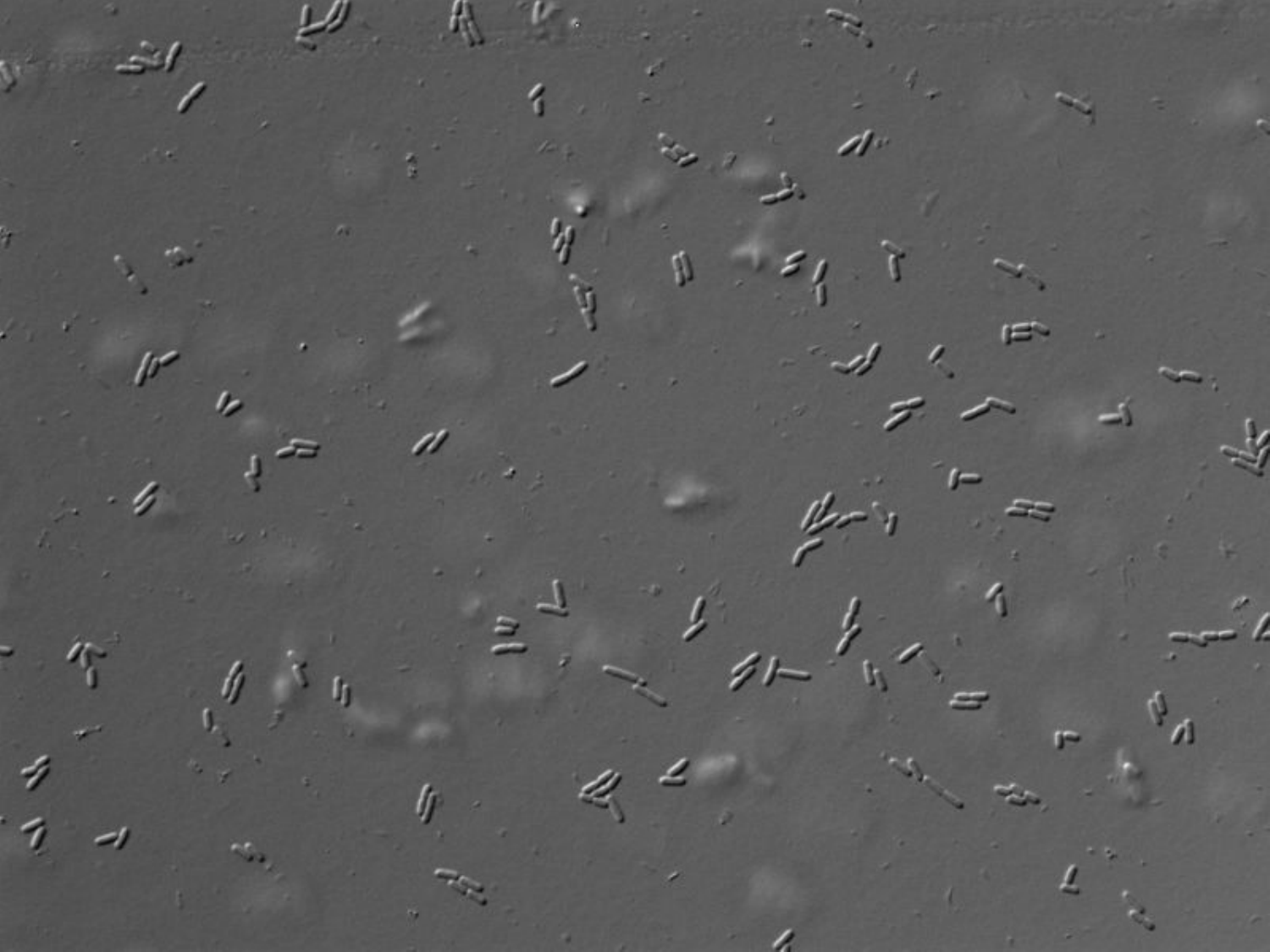

## Slide 6
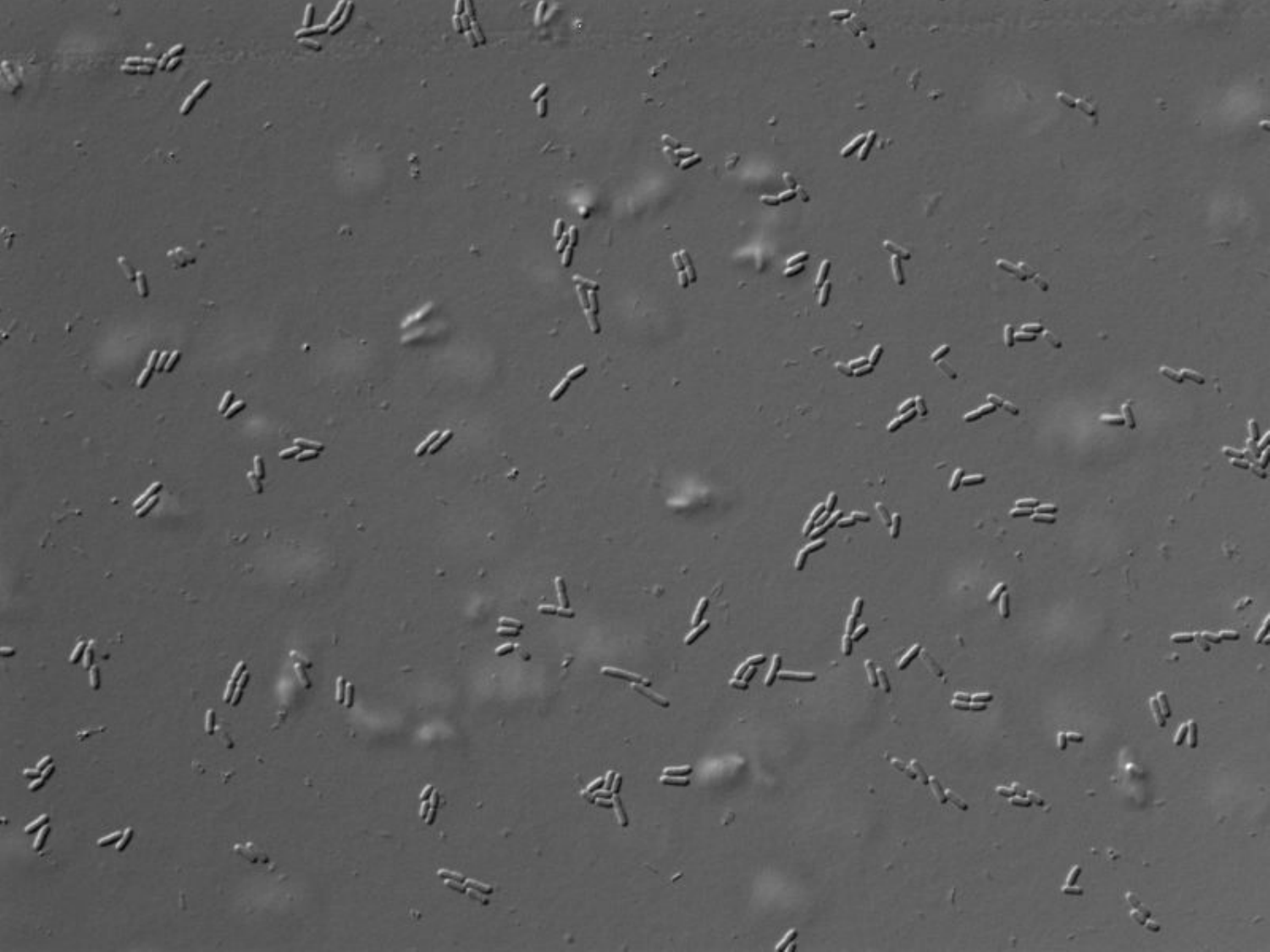

## Slide 7
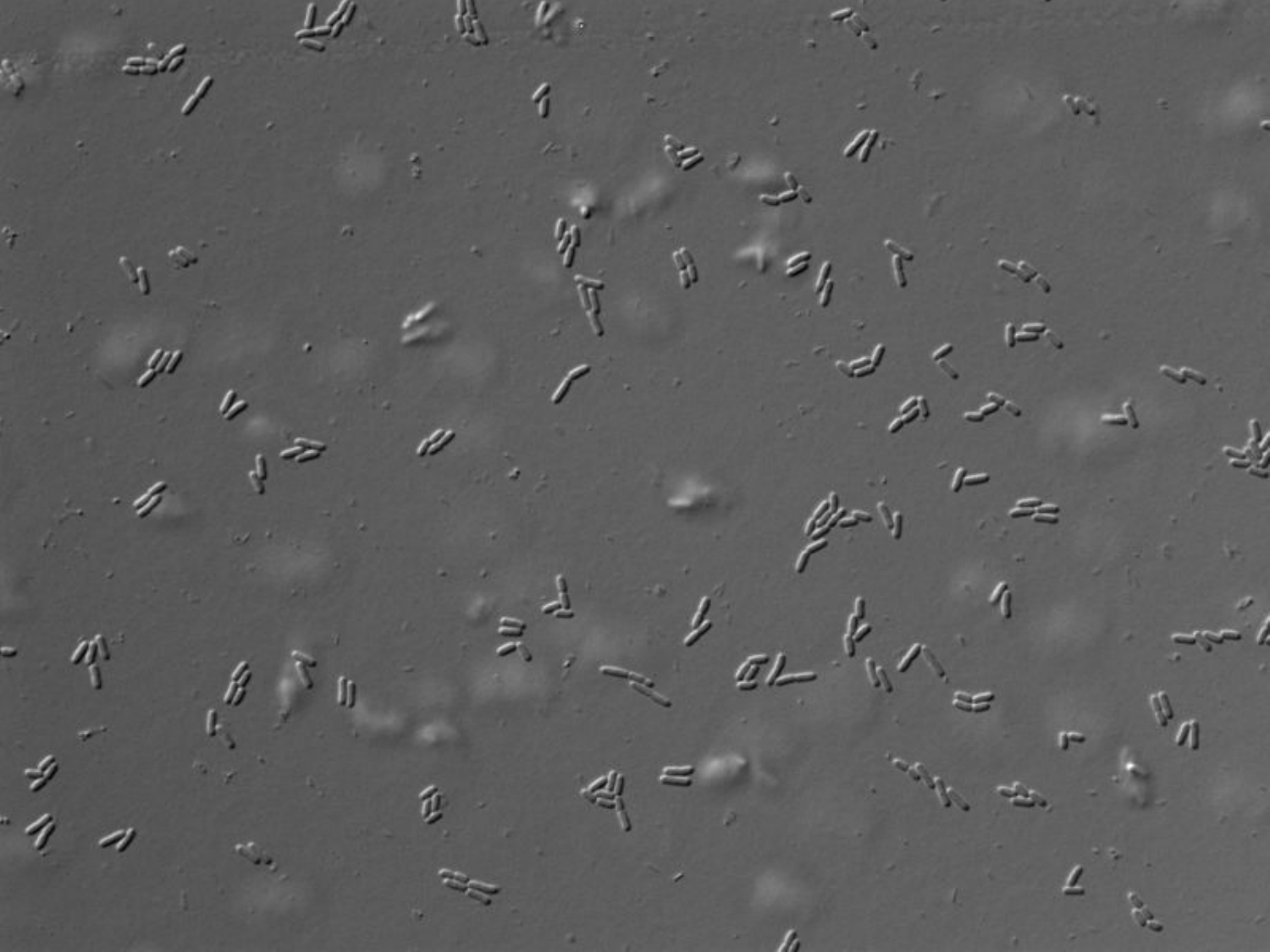

## Slide 8
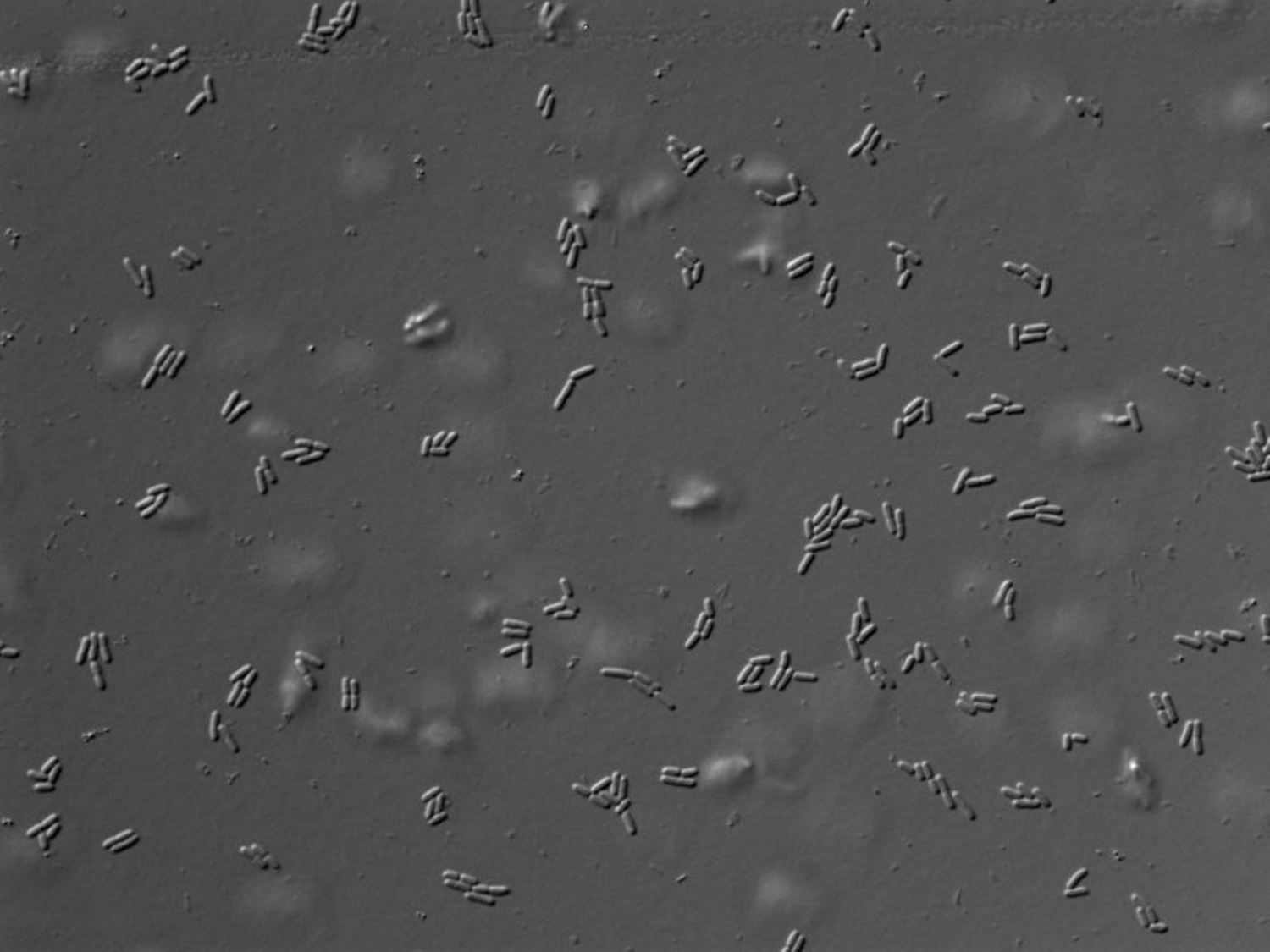

## Slide 9
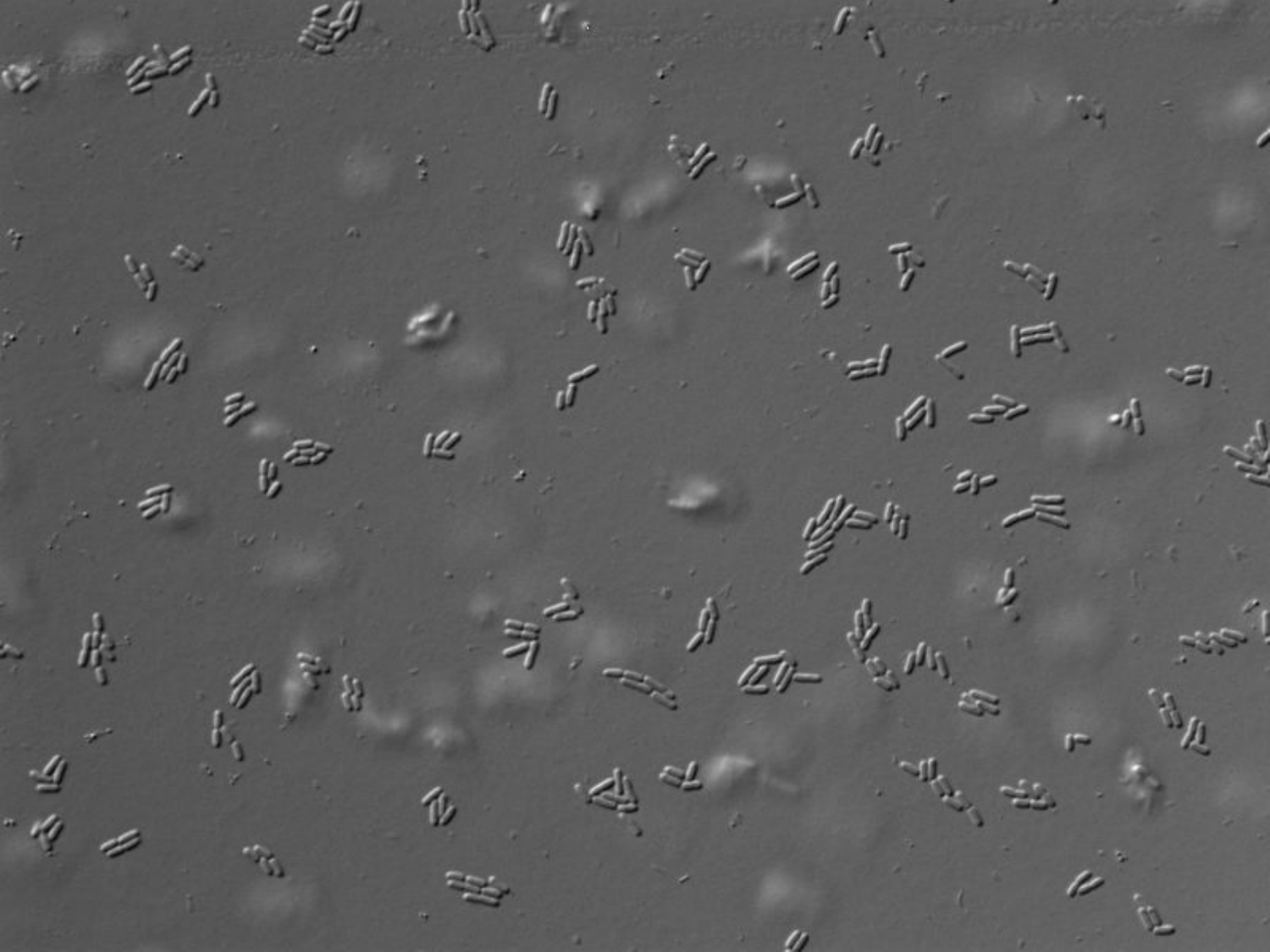

## Slide 10
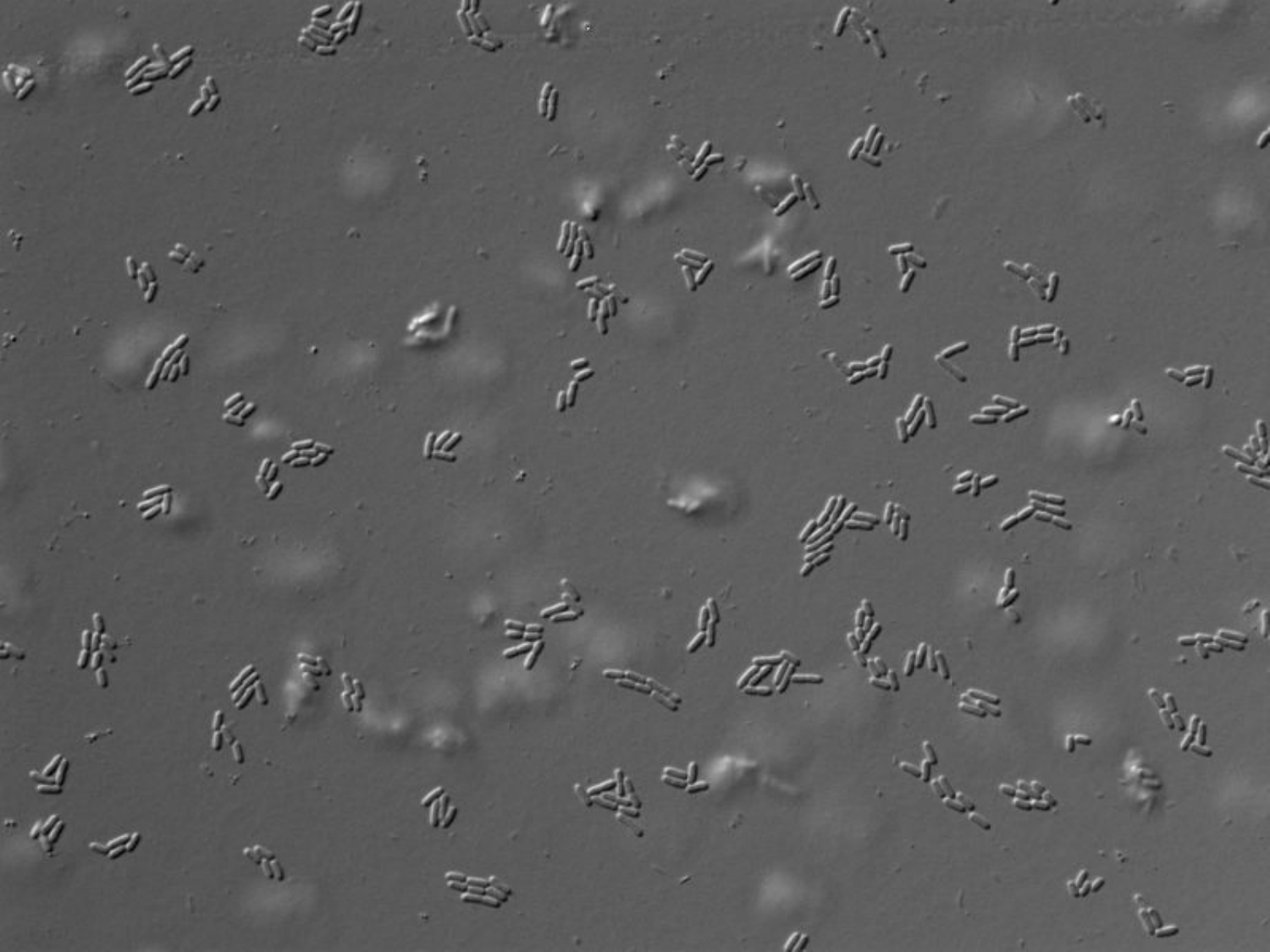

## Slide 11
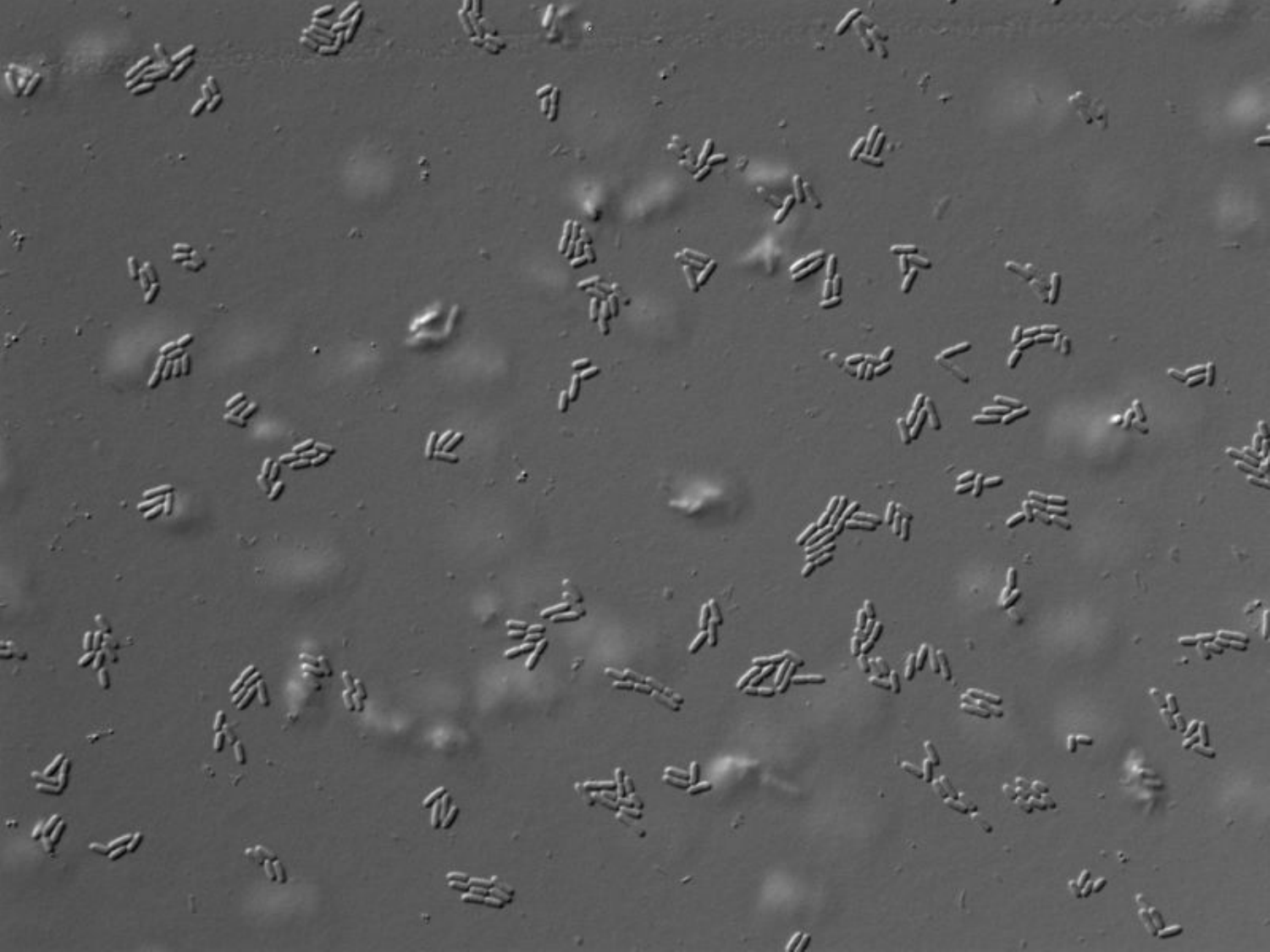

## Slide 12
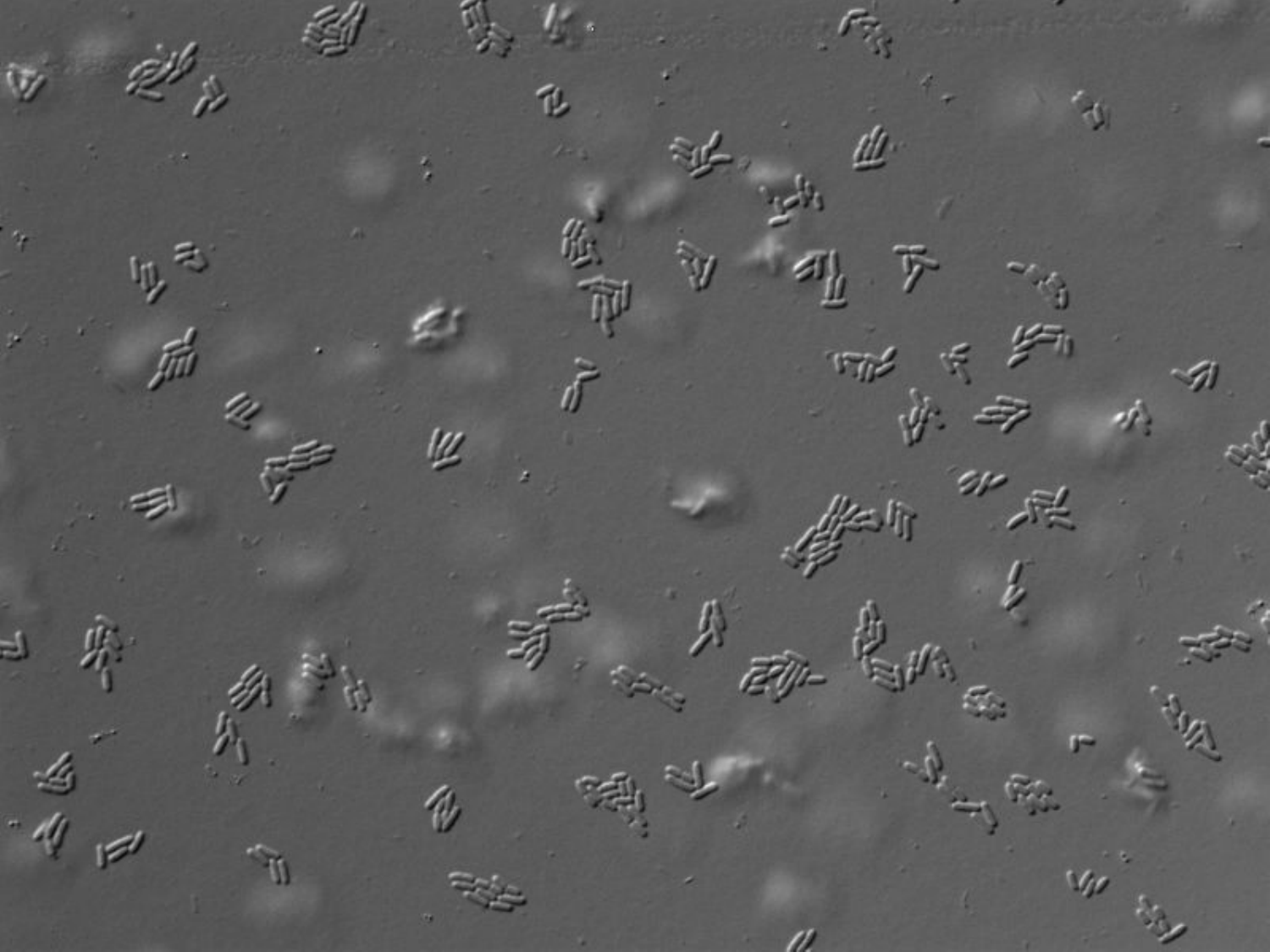

## Slide 13
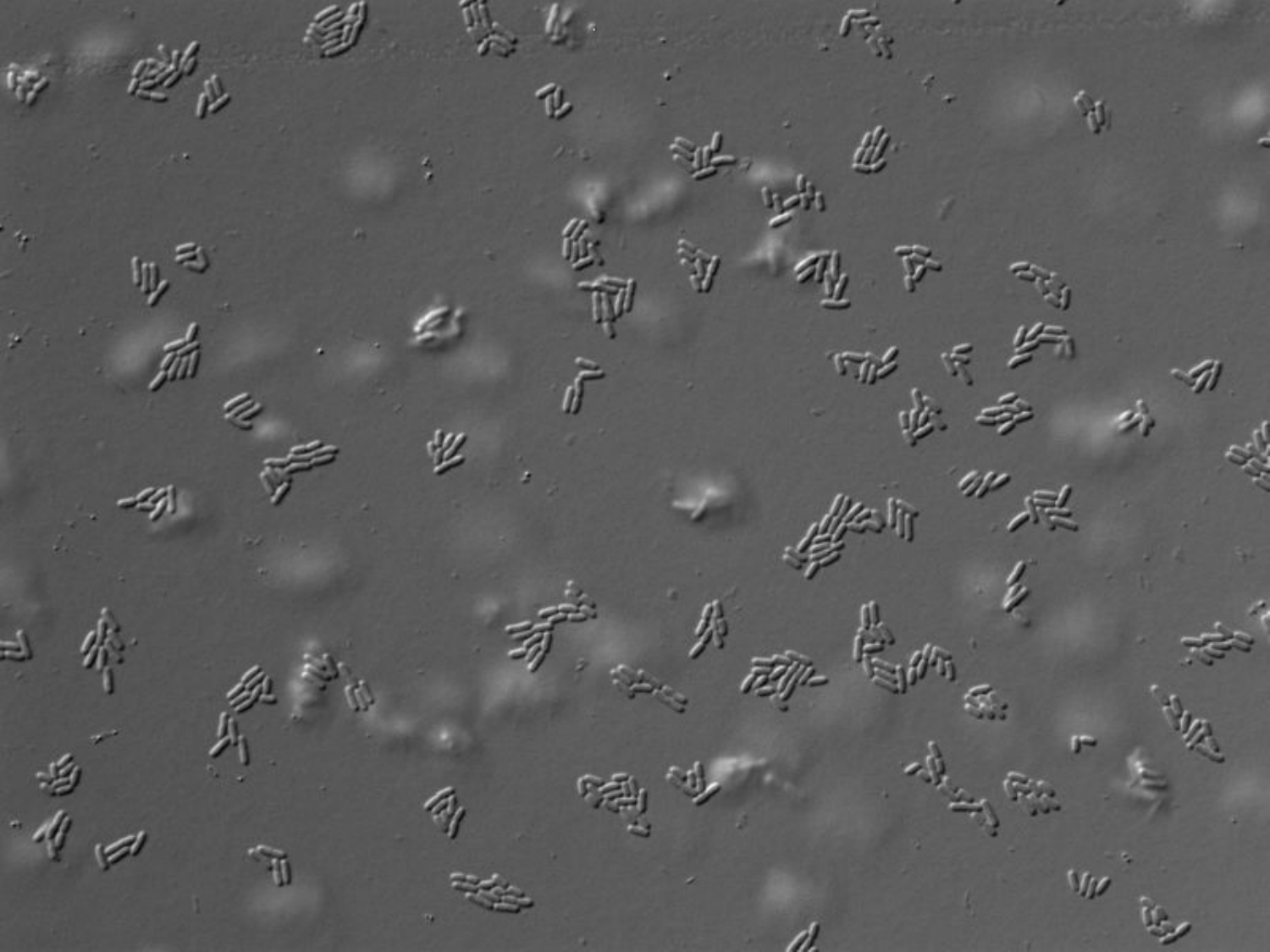

## Slide 14
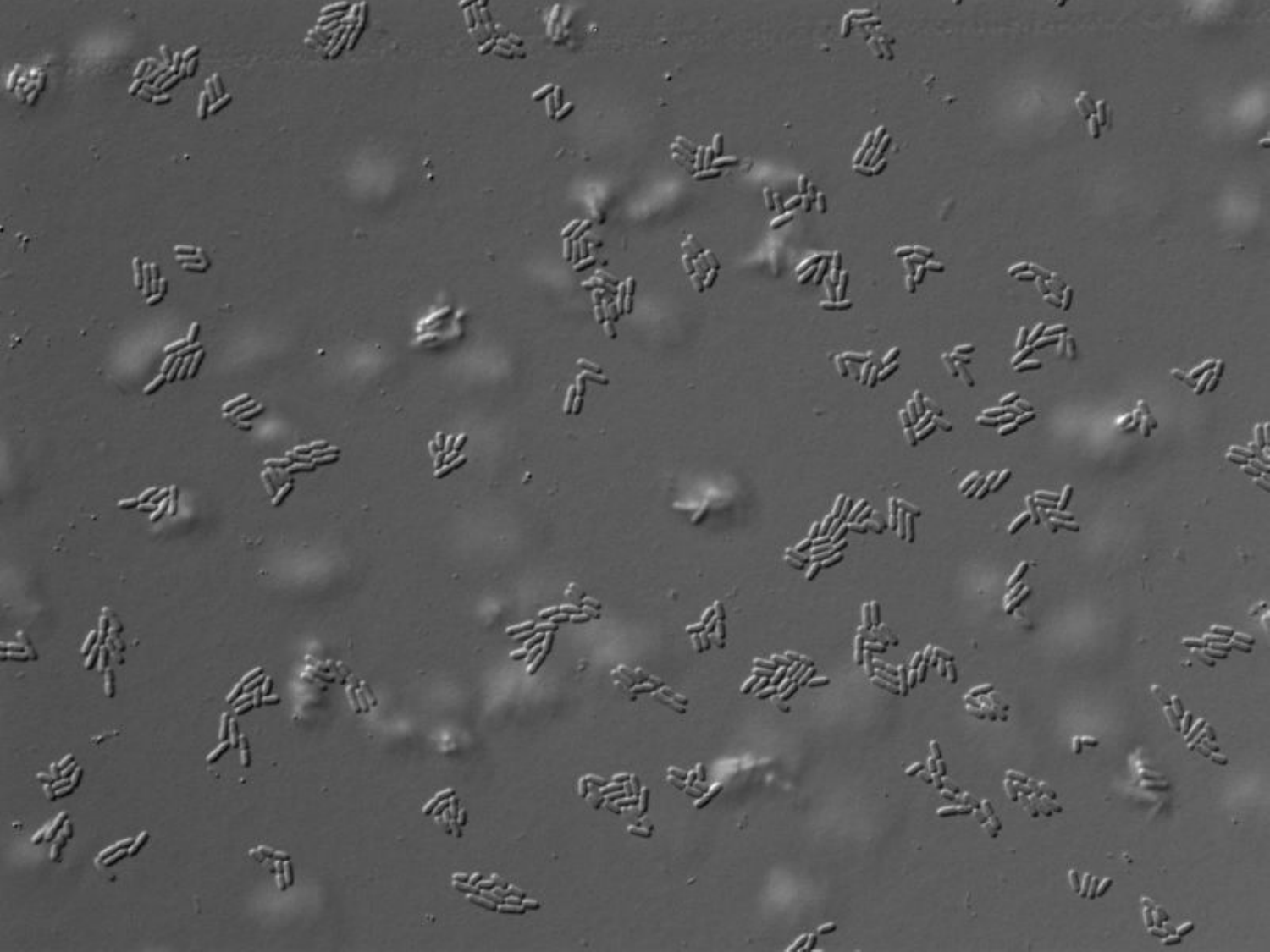

## Slide 15
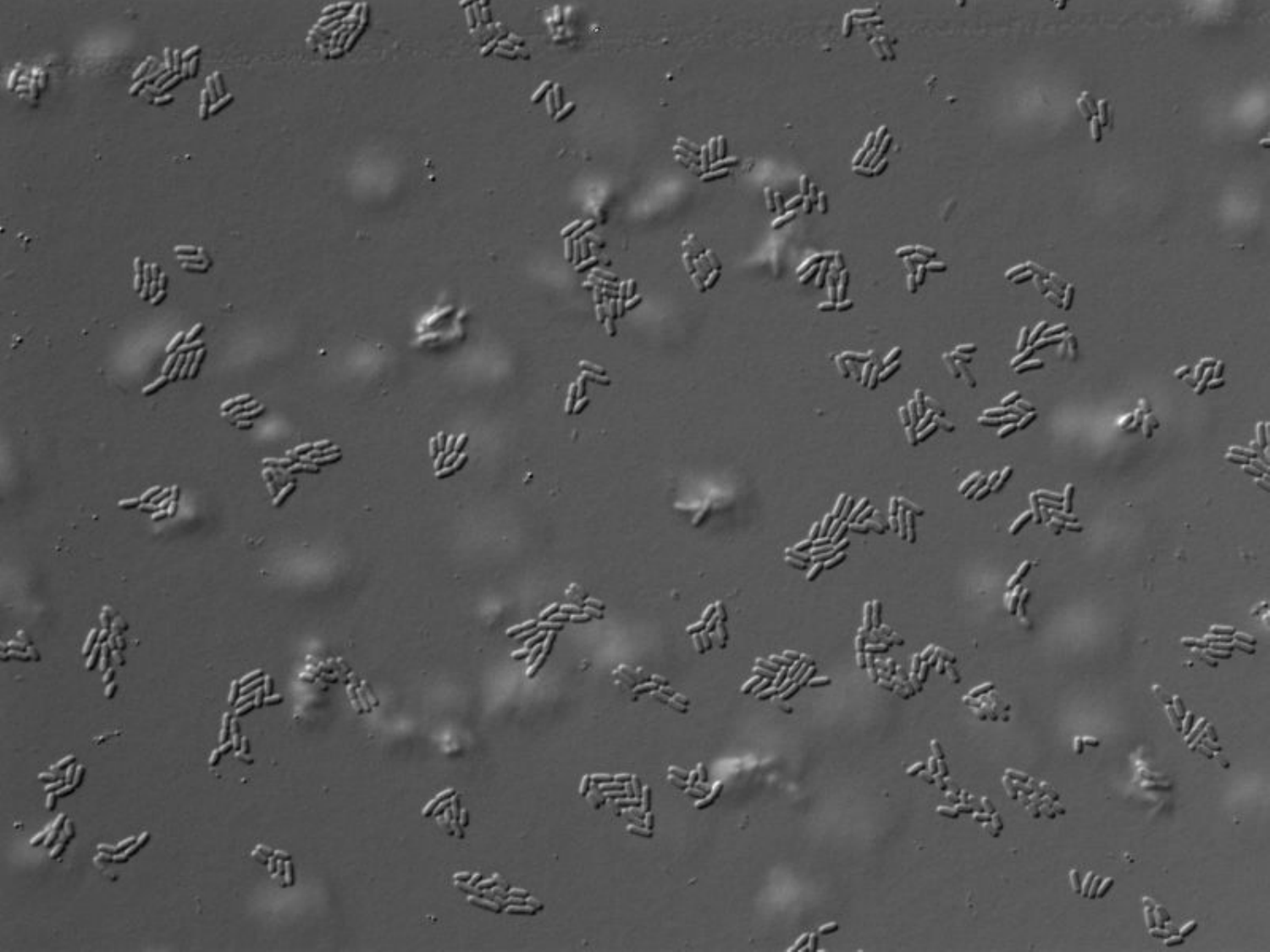

## Slide 16
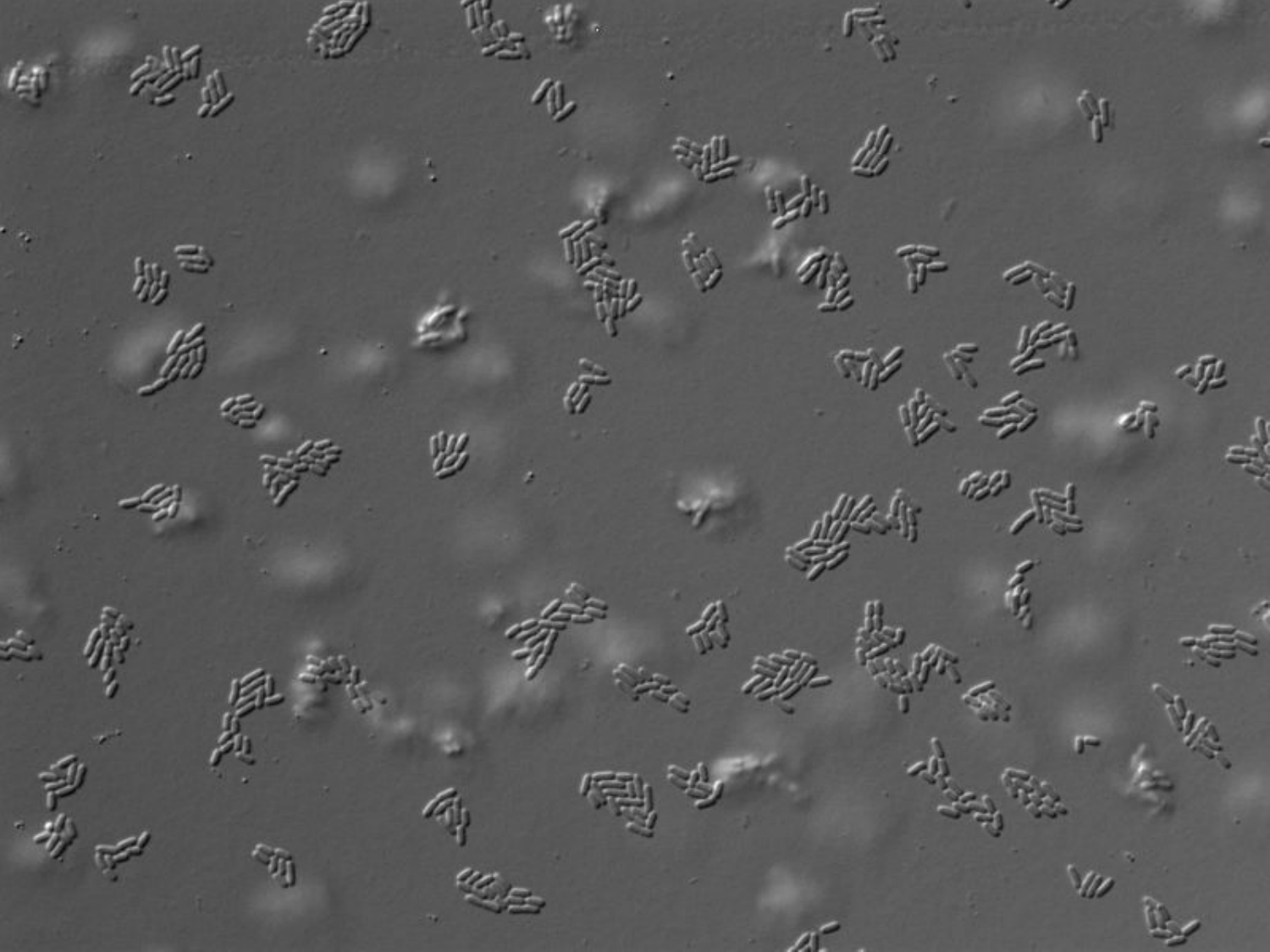

## Slide 17
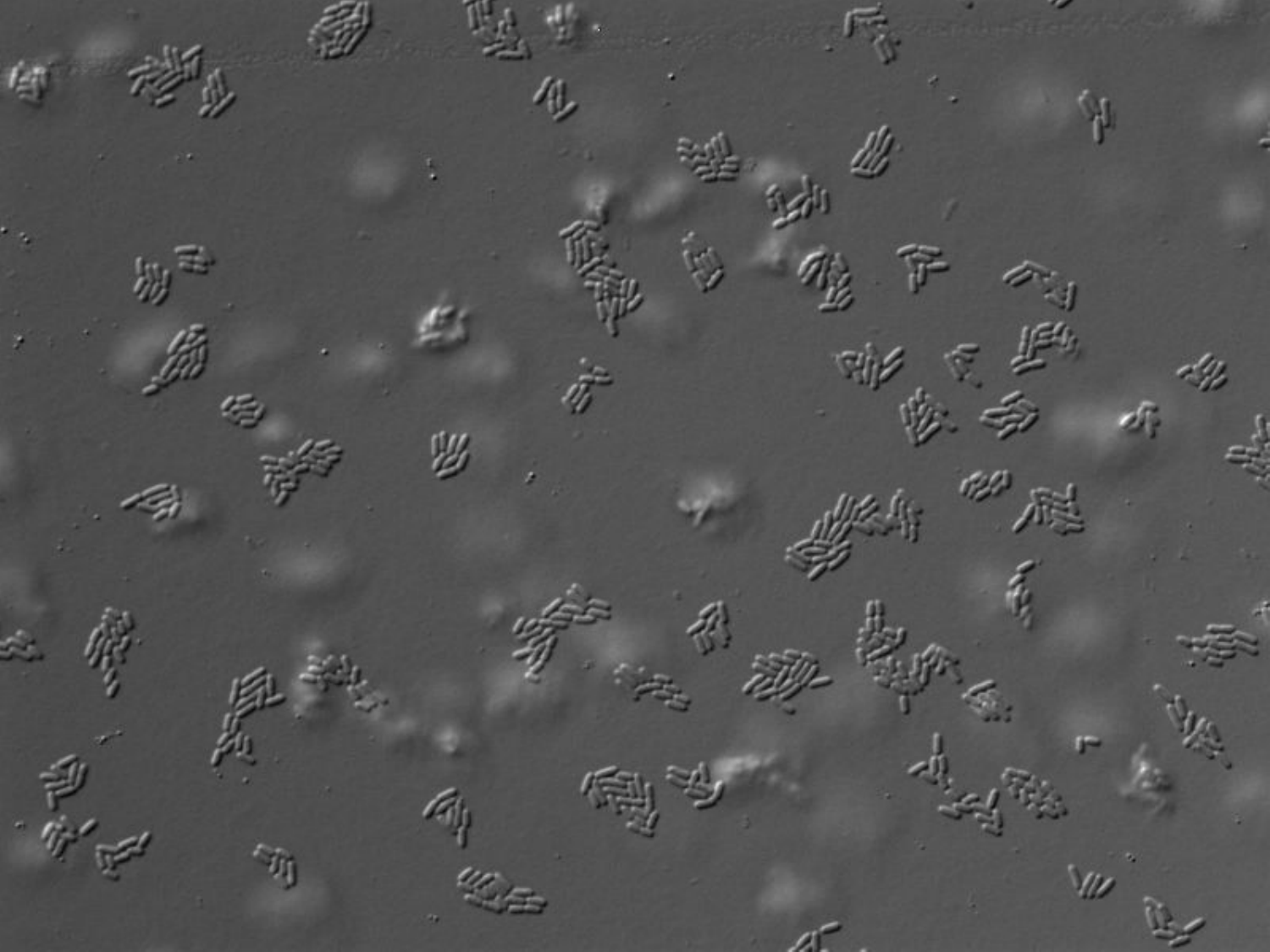

## Slide 18
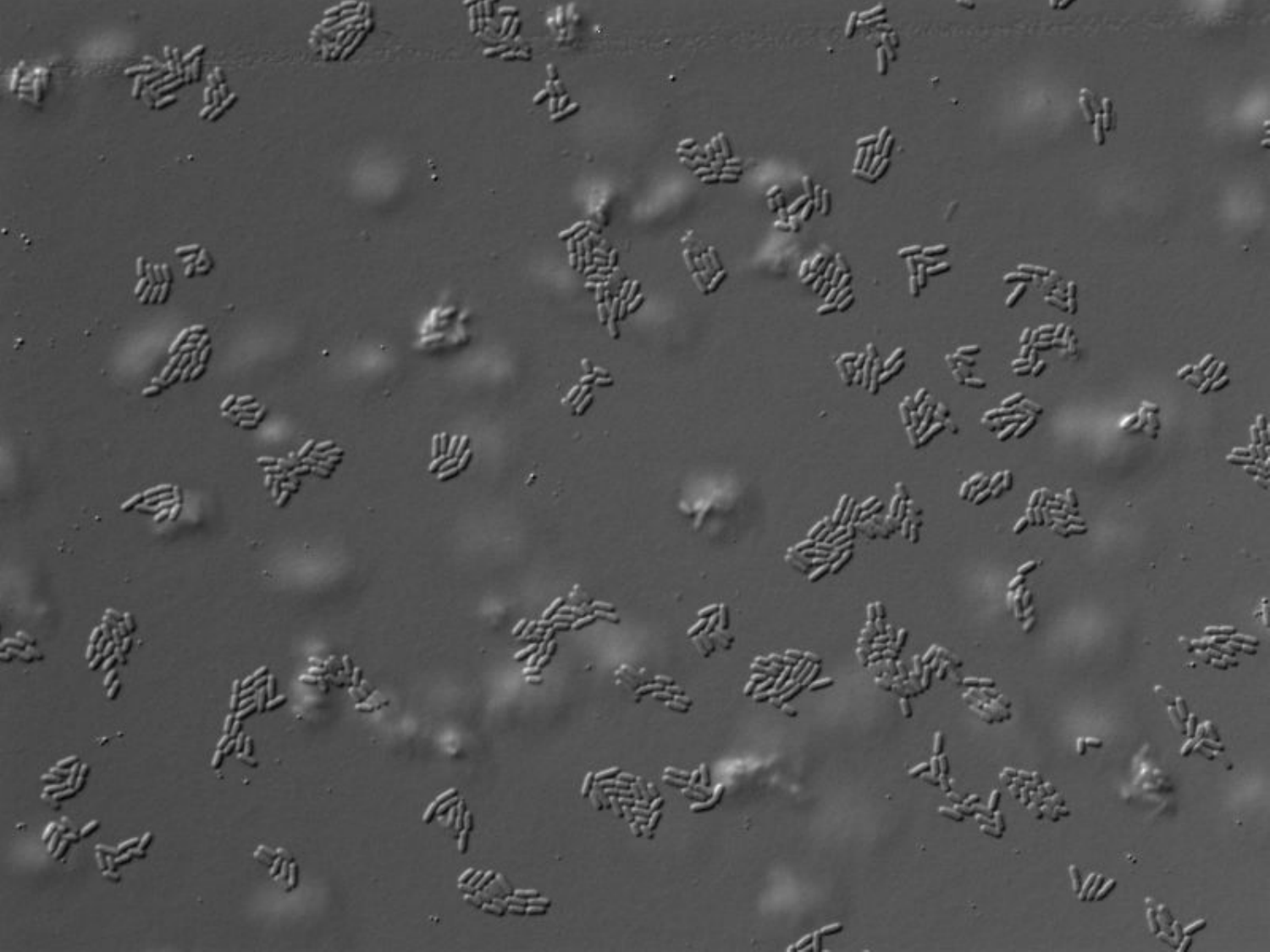

## Slide 19
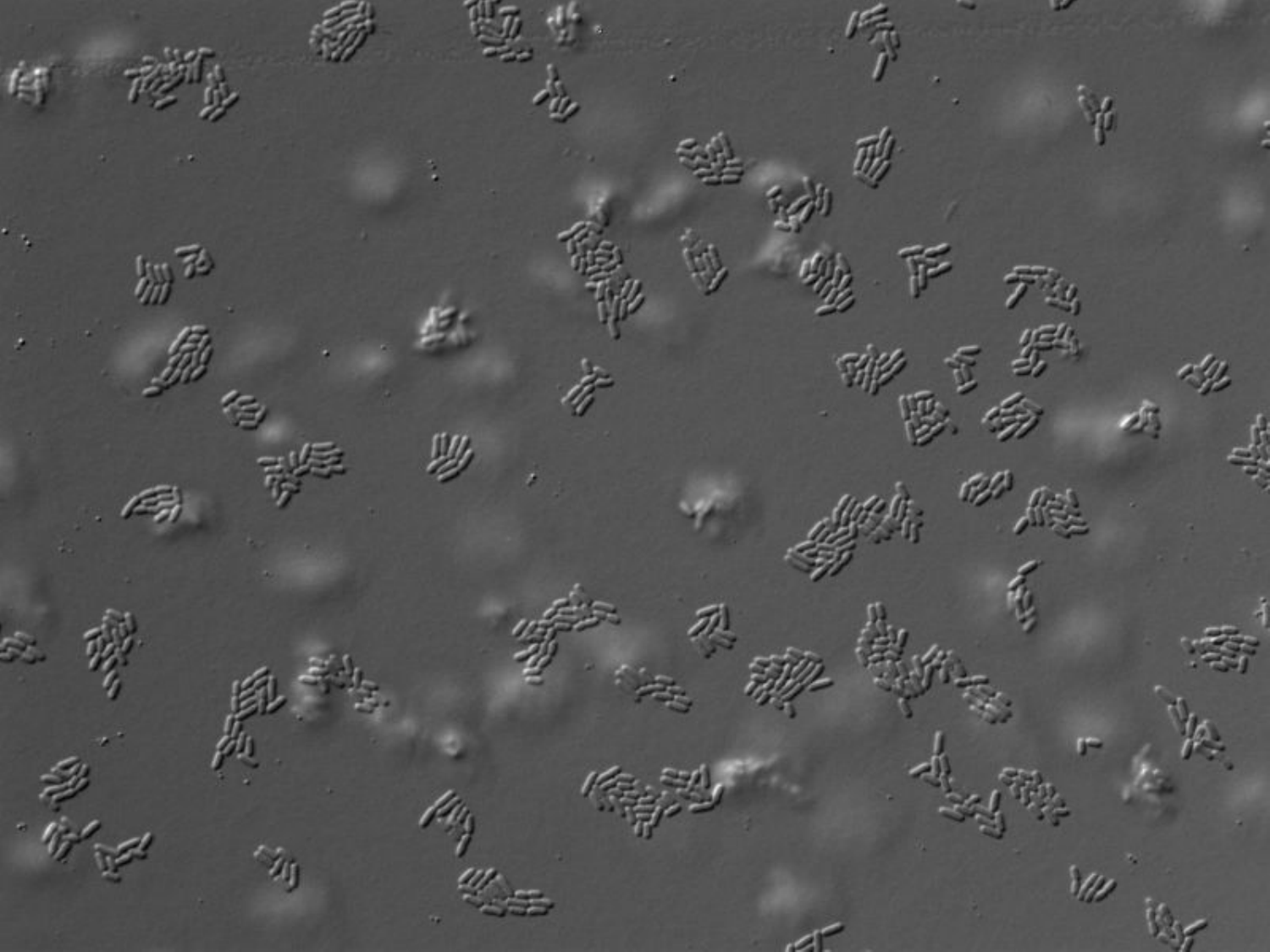

Supplement: Figure S1 — Selected images during microcolony development. (8.05 MB PPT) [file pone.0014516.s004.ppt]
